# Quantification of the dynamics of antibody response to malaria to inform sero-surveillance in pregnant women

**DOI:** 10.1186/s12936-022-04111-y

**Published:** 2022-03-05

**Authors:** A. D. V. Tharkeshi T. Dharmaratne, Saber Dini, Katherine O’Flaherty, David J. Price, James Beeson, Rose McGready, Francois Nosten, Freya J. I. Fowkes, Julie A. Simpson, Sophie G. Zaloumis

**Affiliations:** 1 Centre for Epidemiology and Biostatistics, Melbourne School of Population and Global Health, The University of Melbourne, Melbourne, Australia; 2 Burnet Institute, Melbourne, Australia; 3 Doherty Institute, Melbourne, Australia; 4 Central Clinical School and Department of Microbiology, Monash University, Melbourne, Australia; 5 Department of Infectious Diseases, University of Melbourne, Melbourne, Australia; 6 Shoklo Malaria Research Unit, Mahidol-Oxford Tropical Medicine Research Unit, Faculty of Tropical Medicine, Mahidol University, Mae Sot, Tak, Thailand; 7 Centre for Tropical Medicine and Global Health, Nuffield Department of Medicine, University of Oxford, Oxford, UK; 8 Department of Epidemiology and Preventive Medicine, Monash University, Melbourne, Australia

**Keywords:** Malaria, Pregnancy, Antibodies, *Pf*AMA1, *Pf*EBA175, *Pf*MSP2, *Pf*MSP3, *Pv*AMA1, *Pf*VAR2CSA, Longitudinal data

## Abstract

**Background:**

Malaria remains a major public health threat and tools sensitive to detect infections in low malaria transmission areas are needed to progress elimination efforts. Pregnant women are particularly vulnerable to malaria infections. Throughout pregnancy they access routine antenatal care, presenting a unique sentinel population to apply novel sero-surveillance tools to measure malaria transmission. The aim of this study was to quantify the dynamic antibody responses to multiple antigens during pregnancy so as to identify a single or multiple antibody response of exposure to malaria in pregnancy.

**Methods:**

This study involved a secondary analysis of antibody responses to six parasite antigens [five commonly studied merozoite antigens and the variant surface antigen 2-chondroitin sulphate A (VAR2CSA), a pregnancy-specific erythrocytic antigen] measured by enzyme-linked immunosorbent assay (ELISA) over the gestation period until delivery (median of 7 measurements/woman) in 250 pregnant women who attended antenatal clinics located at the Thai-Myanmar border. A multivariate mixture linear mixed model was used to cluster the pregnant women into groups that have similar longitudinal antibody responses to all six antigens over the gestational period using a Bayesian approach. The variable-specific entropy was calculated to identify the antibody responses that have the highest influence on the classification of the women into clusters, and subsequent agreement with grouping of women based on exposure to malaria during pregnancy.

**Results:**

Of the 250 pregnant women, 135 had a *Plasmodium* infection detected by light microscopy during pregnancy (39% *Plasmodium falciparum* only, 33% *Plasmodium vivax* only and 28% mixed/other species), defined as cases. The antibody responses to all six antigens accurately identified the women who did not have a malaria infection detected during pregnancy (93%, 107/115 controls). Antibody responses to *P. falciparum* merozoite surface protein 3 (*Pf*MSP3) and *P. vivax* apical membrane antigen 1 (*Pv*AMA1) were the least dynamic. Antibody responses to the antigens *P. falciparum* apical membrane antigen 1 (*Pf*AMA1) and *Pf*VAR2CSA were able to identify the majority of the cases more accurately (63%, 85/135).

**Conclusion:**

These findings suggest that the combination of antibodies, *Pf*AMA1 and *Pf*VAR2CSA, may be useful for sero-surveillance of malaria infections in pregnant women, particularly in low malaria transmission settings. Further investigation of other antibody markers is warranted considering these antibodies combined only detected 63% of the malaria infections during pregnancy.

**Supplementary Information:**

The online version contains supplementary material available at 10.1186/s12936-022-04111-y.

## Background

Malaria is a major infectious disease causing around 229 million clinical cases and 409,000 deaths globally in 2019 [1]. Pregnant women are particularly vulnerable to malaria infection, as well as presenting with more severe symptomatic infections [2]. Each year, around 125 million pregnant women, living in malaria endemic countries, are at risk of malaria infection [3, 4]. Malaria in pregnancy poses substantial risks to the pregnant woman and their baby, increasing the risk of maternal anaemia, hypertensive disorders, miscarriage, stillbirth and neonatal death and as such there are several prevention and treatment strategies provided to women attending antenatal care to reduce the burden of malaria in pregnancy [5, 6]. Pregnant women routinely attending antenatal care are also considered an easy-access population which can serve as sentinel surveillance populations to estimate malaria transmission [7].

The development of novel serological surveillance (sero-surveillance) tools for use in sentinel populations of pregnant women is a potential powerful technique for detecting recent and ongoing malaria infections, and monitoring malaria transmission [8, 9]. This is particularly pertinent in low malaria transmission settings such as Southeast Asia, where parasite density is often low and standard surveillance methods (microscopy and rapid diagnostic tests) have reduced sensitivity with low density, submicroscopic and asymptomatic infections [10]. Sero-surveillance tools have the potential to increase the time window for detecting an infection, and thereby increasing the resolution of surveillance [9]. Antibodies targeting blood-stage antigens, predominantly relatively conserved antigens expressed on the merozoites, have been the focus of sero-surveillance studies in non-pregnant populations [9, 11]. In pregnant women, serological studies have also investigated antibody responses to the pregnancy-specific *Plasmodium falciparum* antigen (*Pf*VAR2CSA), which is expressed on the surface of infected erythrocytes (IEs), and mediates sequestration of *P. falciparum* in the placenta via binding to placental chondroitin sulphate A (CSA) receptors [12, 13]. Antibodies specific for *Pf*VAR2CSA may reduce the accumulation of the IEs in the placenta [14]. High antibody levels against *Pf*VAR2CSA can be acquired with successive pregnancies [15], potentially reducing the susceptibility to falciparum malaria in multigravida women by preventing or clearing parasite sequestration in the placenta [16]. While numerous studies have investigated *Pf*VAR2CSA antibodies in pregnant women as markers of infection [17], few studies have incorporated non-pregnancy specific antibodies [18, 19], such as those targeting merozoite antigens, and none have considered the combined effects of these antibodies.

The aim of this study was to quantify the dynamic antibody responses to multiple blood-stage antigens (merozoite and *Pf*VAR2CSA) during pregnancy to determine which, if any, of the antibody response(s) are biomarker(s) of exposure to malaria in pregnancy that could subsequently be used for sero-surveillance. Longitudinal antibody responses to both *P. falciparum* and *Plasmodium vivax* previously measured by ELISA in pregnant women attending antenatal clinics on the Thai-Myanmar border [18], a low malaria transmission setting [20, 21], were jointly analysed to account for the correlations between the antibodies to inform sero-surveillance approaches in pregnant women.

## Methods

### Study population and design

The study population was pregnant women attending antenatal clinics (ANCs) at the Shoklo Malaria Research Unit (SMRU) [22, 23], where malaria transmission is low and peaks between May and September. The ANCs were located in the Maela refugee camps, where approximately 90% of women attend on a weekly basis [23].

**Fig. 1:**
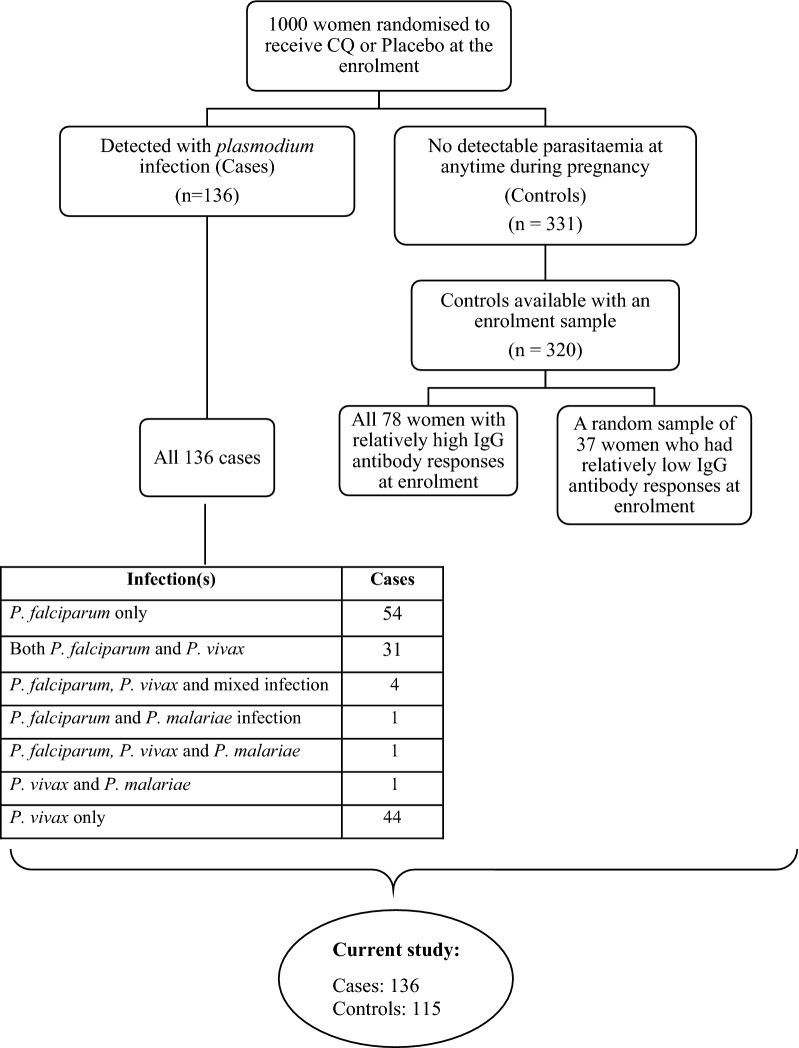
Flow diagram of cohort selection for the current study

Details of the nested case–control study design and procedures have been published previously [18]. In brief, participants were identified from 1000 Karen women who were enrolled in a placebo randomized controlled trial of chloroquine prophylaxis against *P. vivax* infection during pregnancy from November 1998 through January 2000 [24]. Samples were obtained weekly from the women for *Plasmodium* species infection detection by microscopic examination of blood smears and fortnightly for serum sample collection. Case subjects were women with *Plasmodium* infection detected by light microscopy at any time during pregnancy during the trial (n = 136). Of the 864 women with no detectable parasitaemia at any time while pregnant during the trial, 331 were randomly selected to be control subjects (3:1 ratio). All available serum samples from the 136 case subjects were included for quantification of antibodies to multiple antigens over time [18]. A subset of 115 control subjects was selected for longitudinal antibody determination based on IgG responses to *P. falciparum* (3D7) schizont extract measured by ELISA at enrollment. The 115 controls were selected as follows. All available control enrollment samples (320 of the 331 randomly selected controls had serum samples measured at enrollment) were first tested for total IgG in response to *P. falciparum* schizont protein extract. A cut-off threshold for seropositivity to schizont extract was set to the mean + 3 standard deviations of the IgG responses to schizont extract for 8 negative controls (non-exposed Melbourne donors). The subset of 115 controls consisted of 78 individuals seropositive to schizont extract at enrollment (total of n = 572 samples included) together with 37 randomly selected individuals who were seronegative to schizont extract at enrollment (total of n = 323 samples included) (Fig. 1). See Additional file 1, Fowkes et al. [18] for further details. Of note, the nested case–control study design for the selected serum samples was not designed for this analysis.

### Antibody determination

High throughput enzyme-linked immunosorbent assay (ELISA) [18, 25] was used to determine the total IgG magnitude (measured as optical density (OD) values) of *P. falciparum* merozoite antigens and one *P. vivax* merozoite antigen (apical membrane antigen-1 [*Pv*AMA1] ectodomain based on the SalI sequence, PVX_ 092275) [26]. *P. falciparum* antigens included were (from 3D7 reference sequence): apical membrane antigen 1 (*Pf*AMA1; ectodomain) [27], erythrocyte binding antigen 175 (*Pf*EBA175; region 3–5) [28], merozoite surface protein 2 (*Pf*MSP2, full length) [27], merozoite surface protein 3 (*Pf*MSP3; amino acids 154–249) [29, 30], schizont extract, *Pf*VAR2CSA (DBL5ɛ domain), (Additional file 1: Supplementary methods and Fowkes et al. [18]). Schizont lysate was prepared from *P. falciparum* (3D7) cultures according to previously described methods [27].

### Statistical analysis

Patient characteristics at baseline were summarized using median (25th–75th percentiles) for continuous variables or frequency (%) for categorical variables.

A multivariate mixture linear mixed model was used to identify clusters (i.e., latent classes) of pregnant women that have similar antibody responses to all six antigens over gestational age [31]. To construct a multivariate mixture linear mixed effects model, first a linear mixed effects model was specified for each of the six antibody responses. The following covariates were included as fixed effects for each antibody response: age (years), primigravidae (1 if primigravidae and 0 if multigravida), treatment arm (1 if given chloroquine (CQ) as prophylaxis at enrolment and 0 if given a placebo) and having a history of malaria prior to enrolment (1 if exposed to malaria at least once prior to enrolment and 0 otherwise). To capture the between-subject variability in the six antibody responses over gestational age, a random intercept and random slope for gestational age were included in the linear mixed effects model for each antibody response. The random intercepts and slopes for each response and woman (i.e., 12 random effects per woman) are assumed to follow a mixture of multivariate normal distributions.

The mixAK package in R [32] was used to fit the multivariate mixture linear mixed effects model to the six antibody response profiles available from each of the 250 women (135 cases and 115 controls) in this study. The mixAK package adopts a Bayesian approach to inference and implements a block Gibbs sampler with Metropolis–Hastings steps to sample parameter values from the posterior distribution (Additional file 1, Komárek and Komárková (2013, Appendix B) [31]). Weakly informative prior hyperparameters in the mixAK package were used (Additional file 1, Komárek and Komárková (2013, Appendix A) [31]). Two chains were initialized. The first 500 parameter values sampled for each chain were discarded as burn-in and an additional 5,000,000 parameter values (in total for both chains) were sampled after burn-in. Every 50th iteration after burn-in was kept, resulting in 100,000 (50,000 per chain) samples per parameter for calculation of posterior summaries. Results are presented as the posterior median (50th percentile) and 95% credible interval, calculated as the 2.5th and 97.5th percentiles of the 100,000 samples for each parameter. Traceplots were examined to assess whether the 50,000 parameter draws from each chain had appropriately converged.

The number of clusters was selected by fitting a mixture model assuming each of 1–4 clusters. The number of clusters was selected according to the model that produced the lowest penalized expected deviance and/or greatest shift of the posterior distribution of deviances to lower values [31]; Additional file 1: Supplementary methods, Statistical Analyses.

The posterior probability of a woman belonging to a cluster (posterior class probability) was calculated at each iteration of the fitting algorithm and these probabilities were used to assign a woman to a cluster as follows. First, a woman was assigned to the cluster which had the highest median posterior class probability. Second, the woman remained in the cluster if the lower limit of the 95% credible interval for the posterior class probability exceeded 0.5; otherwise, the woman was considered unclassified (Additional file 1: Supplementary methods, Statistical Analyses).

The variable-specific entropy was calculated to identify the antibody responses that have the highest influence on the classification of the women into clusters. The variable-specific entropy indicates how well the antibody response to a single antigen predicts the classification based on antibody responses to all six antigens, and ranges from 0 to 1 [33]. Antibody responses with variable-specific entropy values close to 1 drive the classification of women into clusters (Additional file 1: Supplementary methods, Statistical Analyses).

To compare and identify the best antibodies for classifying women as being a case (defined as malaria detected by microscopy during pregnancy) or control, additional univariate and pairwise multivariate mixture linear mixed-effects modelling were performed with the number of clusters set at two groups, and the proportion of cases and controls classified in the high and low antibody response groupings calculated.

## Results

### Patient characteristics

A total of 1692 samples were included in the analysis; 727 samples were available from the 135 cases (1 case excluded because only an enrolment sample was available) and 965 from the 115 controls. For the 135 cases, 39% had *P. falciparum* infections only, 33% *P. vivax* only and 28% mixed/other species, see Additional file 1: Table S1 for number and species of infection(s) recorded during pregnancy.

Table 1 presents the baseline characteristics of the 135 cases (women with *P. falciparum* and/or *P. vivax* detected by microscopy during pregnancy) and 115 controls (women without detected *Plasmodium* infection). The proportion of cases in their first pregnancy (22.2%) at enrolment was around twice of that in the control group (13.9%). Anaemia was more prevalent among cases at enrolment (23.7%), compared to controls (9.6%). The proportion of women who had received chloroquine prophylaxis (only to prevent vivax episodes) [24] was slightly higher for the control group (47.8% vs 41.5%). For both cases and controls, most women were enrolled in the trial during their first trimester (cases 75.6% and controls 85.2%). Nearly all controls were residing in refugee camps (99.1%), while less than half of the cases (45.2%) were living in refugee camps during pregnancy (the remaining cases were residing in villages south of the Mae Sot township).

**Table 1:** Distribution of patient characteristics at enrollment for cases and controls

Characteristic	Cases (n = 135)	Controls (n = 115)
Age (years), median (IQR)	24 (20–30)	26 (22–32)
Gravidity, median (IQR)	3 (2.0–4.5)	3 (2.0–5.0)
Primigravida, n (%)	30 (22.2)	16 (13.9)
Multigravida, n (%)	105 (77.8)	99 (86.1)
Parity, median (IQR)	1 (0–3)	2 (1–4)
Haematocrit (%), median (IQR)	32.5 (30–35)	34.0 (32–36)
Anaemia^a^, n (%)	32 (23.7)	11 (9.6)
Residence in refugee camp	61 (45.2)	114 (99.1)
Received chloroquine prophylaxis, n (%)	56 (41.5)	55 (47.8)
Estimated Gestational Age^b^(weeks), median (IQR)	9.7 (7–14)	9.4 (7.6–11.6)
Trimester		
1 (< 14 weeks), n (%)	102 (75.6)	98 (85.2)
2 (14 to < 28 weeks), n (%)	31 (23)	17 (14.8)
3 (28 week or more), n (%)	2 (1.5)	0 (0.0)
* Plasmodium* spp. before enrolment^c^, n (%)	75 (55.6)	42 (36.5)
Infected with *P. falciparum*^d^, n (%)	50 (37.9)	32 (27.8)
Infected with *P. vivax*^d^, n (%)	32 (24.2)	13 (11.3)
Follow up (weeks), median (range)	28.9 (22.6–32.1)	30.9 (28.3–32.4)

^a^Haematocrit < 30%

^b^Determined at enrolment

^c^Any microscopically confirmed *Plasmodium* infection documented at SMRU before enrolment into the study

^d^Includes women that have a history of both *P. falciparum* and *P. vivax* infections. Hence, the summation of *P. falciparum*^d^ and *P. vivax*^d^ women does not add up to *Plasmodium* spp. before enrolment^c^

More than half of the cases (55.6%) had a documented history of malaria at the SMRU ANCs (any *Plasmodium spp.*; 37.9% *P. falciparum* and 24.2% *P. vivax*) prior to enrollment compared with 36.5% of the controls (27.8% *P. falciparum* and 11.3% *P. vivax*).

### Antibody dynamics over gestation

**Fig. 2:**
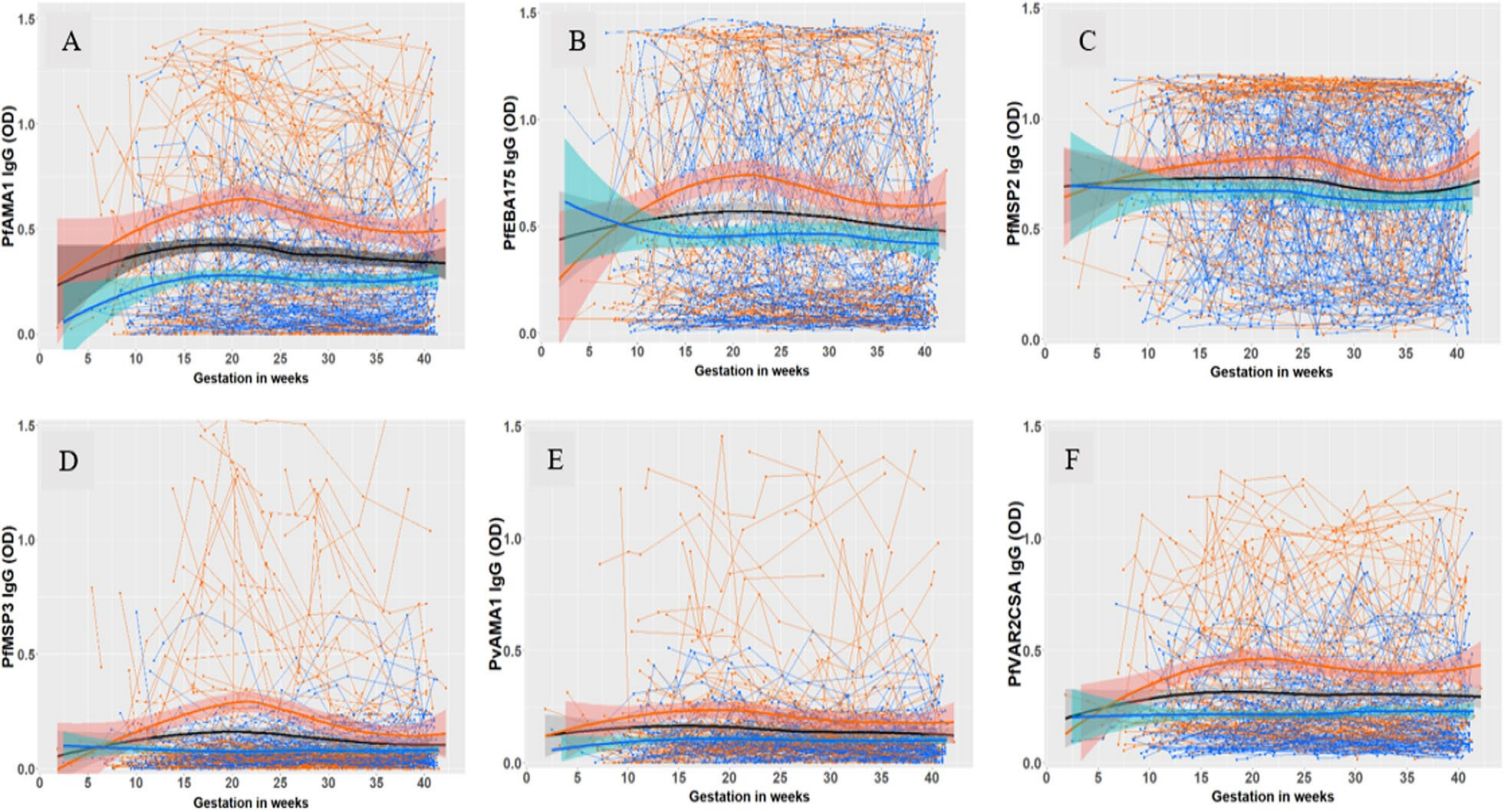
Longitudinal antibody levels against six antigens for all women. Spaghetti plots **A**–**F** represent the antibody profiles of *Pf*AMA1, *Pf*EBA175, *Pf*MSP2, *Pf*MSP3, *Pv*AMA1 and *Pf*VAR2CSA, respectively. The antibody levels of the pregnant women exposed to malaria (cases) and free from malaria by microscopy (controls) are represented by orange and blue, respectively. Locally estimated scatterplot smoothing (LOESS) curves for all pregnant women (in black) and for each exposure group are superimposed on each spaghetti plot. The shaded area around each LOESS curve represents the 95% confidence interval (CI). Of note, the Y axes of the plots of **A**, **B** and **D** are truncated at 0; the CIs did extend to negative values due to limited information in the early period of gestation. *OD* optical density

For all six antigens, the antibody response profiles of pregnant women who were infected with malaria during the trial (cases) tended to be maintained at higher levels compared to women who were free from malaria (controls) (Fig. 2). However, substantial variation in the antibody profiles of *Pf*AMA1, *Pf*EBA175, *Pf*MSP2 and *Pf*VAR2CSA was observed, irrespective of exposure to malaria (Fig. 2A–C, F). For *Pf*MSP3 and *Pv*AMA1, the antibody profiles tended to remain low over gestation for most controls, while the antibody responses for the cases exhibited greater variability (Fig. 2D–E). The controls consisted of 78 individuals’ seropositive to schizont extract at enrolment together with 37 randomly selected individuals who were seronegative to schizont extract at enrolment. Additional file 1: Fig. S1 shows that the longitudinal antibody responses of these two control subgroups are similar, with slightly higher values in *Pf*AMA1 and *Pf*MSP2 for those seropositive to schizont extract.

The behaviour of the longitudinal antibody profiles by gravidity (primigravidae versus multigravidae) of the pregnant women are presented in Additional file 1: Fig. S2, and suggest similar between- and within-individual variation in levels for primigravid and multigravida women.

### Classification of pregnant women

**Fig. 3:**
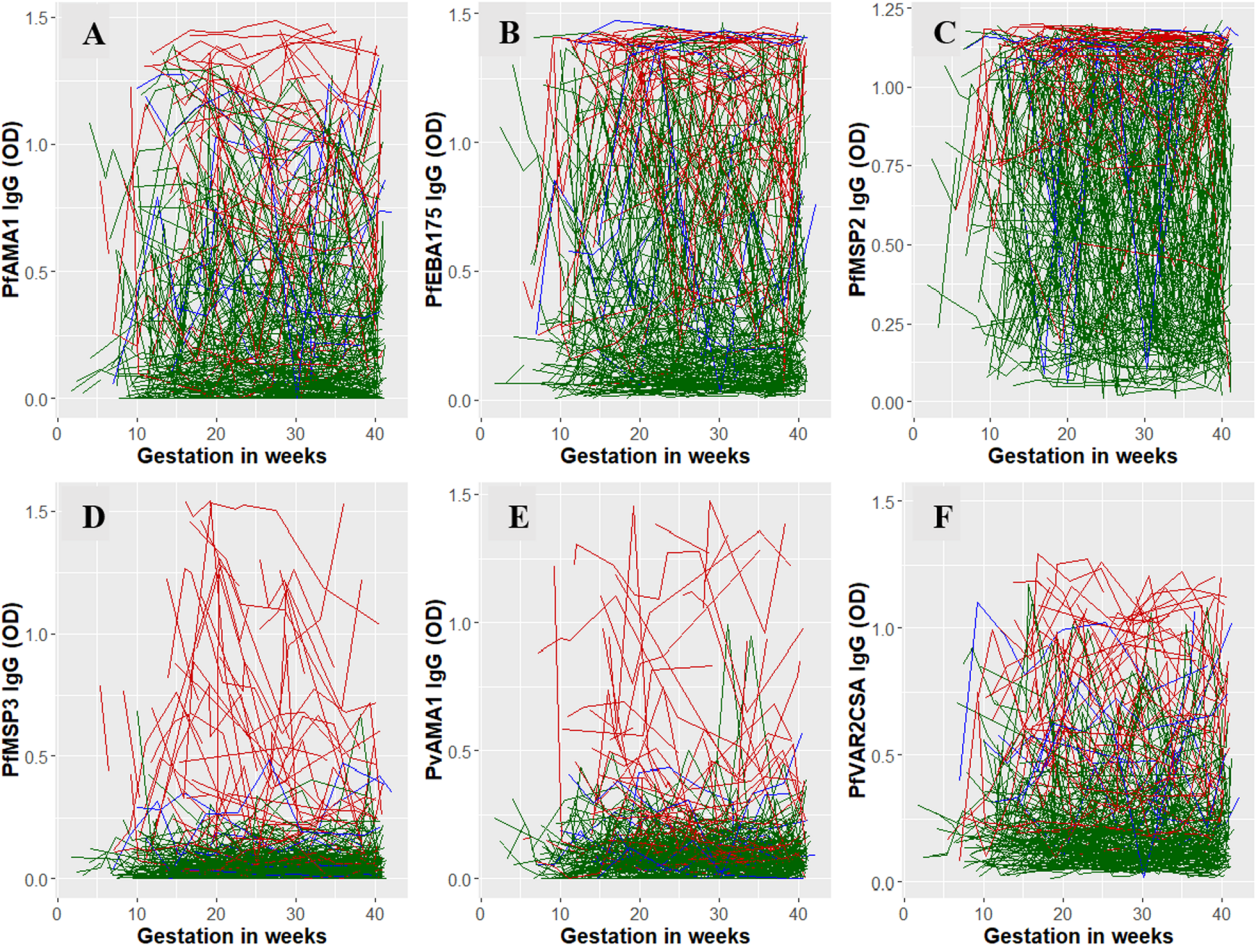
Trajectory plot of the antibody responses versus gestation (weeks) for each individual by cluster (cluster 1 (low immune)—green, cluster 2 (high immune)—red). The trajectories in blue are the pregnant women who were not classified with certainty into either cluster. *OD* optical density

The best fitting multivariate mixture linear mixed effects model according to the penalized expected deviance and the posterior distribution of the deviances classified the six antibody profiles from each of the 250 pregnant women into two clusters (Additional file 1: Supplementary methods, Sects. 1–5): 186 into Cluster 1 and 55 into Cluster 2, with 9 unclassified (see Additional file 1: Table S3 for baseline characteristics of pregnant women by cluster group). The antibody response profiles allocated to each cluster are shown in Fig. 3 and demonstrate that the clustering method has clearly differentiated Cluster 1 as a group of pregnant women with antibody profiles maintained at relatively low levels or dynamic low-medium levels across all 6 antibodies, from Cluster 2 as those who had relatively high or dynamic medium–high antibody levels over gestational age. As such, herein clusters 1 and 2 are referred to as “low immune” and “high immune” groups, respectively.

### Association between maternal factors and antibody responses

Posterior summaries for the fixed effect and population average intercept and slope parameters of the multivariate mixture linear mixed effects model are presented in Table 2. For most antigens, the mean change in antibody responses increased with age (per year) and were higher for those pregnant women that received chloroquine prophylaxis; the exceptions were the mean change in antibody titer to *Pv*AMA1 decreased by − 0.001 with age (per year) (95% Credible Interval (CrI): − 0.002, 0.0004)) and the mean antibody titer to *Pf*MSP3 were − 0.003 (95% CrI: − 0.019, 0.013) lower for those that received chloroquine prophylaxis compared to those that did not. Mean antibody responses to *Pf*AMA1, *Pf*MSP2 and *Pf*MSP3 were increased for primigravidae compared to multigravidae women. For pregnant women with a history of malaria, only mean antibody responses to the antigen *Pv*AMA1 were increased (0.012 (95% CrI: − 0.005, 0.028)) compared to women without a history of prior infection(s). Of note, the majority of the limits of the 95% CrIs ranged from decreasing to increasing changes in mean antibody responses so the associations with the maternal factors described above are weak.

**Table 2:** Multivariate linear mixed-effects modelling of the combined IgG responses (measured as optical density (OD) values) to *Plasmodium falciparum* and *Plasmodium vivax* recombinant antigens

Variable	*Pf*AMA1	*Pf*EBA175	*Pf*MSP2	*Pf*MSP3	*Pv*AMA1	*Pf*VAR2CSA
Fixed effects^a^						
Age (years)	0.001 (− 0.006, 0.008)	0.007 (− 0.001, 0.015)	0.005 (− 0.001, 0.01)	0.001 (0.0001, 0.003)	− 0.001 (− 0.002, 0.0004)	0.003 (− 0.0001, 0.007)
Gravidity						
Primigravidae	0.064 (− 0.05, 0.179)	− 0.017 (− 0.153, 0.118)	0.013 (− 0.075, 0.102)	0.004 (− 0.018, 0.027)	− 0.005 (− 0.029, 0.02)	− 0.012 (− 0.069, 0.046)
Intervention group						
Chloroquine	0.054 (− 0.021, 0.13)	0.048 (− 0.041, 0.136)	0.009 (− 0.05, 0.067)	− 0.003 (− 0.019, 0.013)	0.019 (0.002, 0.036)	0.031 (− 0.007, 0.069)
History of malaria						
Yes	− 0.04 (− 0.116, 0.036)	− 0.024 (− 0.113, 0.065)	− 0.02 (− 0.08, 0.039)	− 0.002 (− 0.018, 0.013)	0.012 (− 0.005, 0.028)	− 0.014 (− 0.053, 0.024)
Average random effects for a woman						
Low immune						
Mean antibody level^b^	0.31 (0.231, 0.388)	0.469 (0.373, 0.564)	0.704 (0.631, 0.777)	0.069 (0.048, 0.09)	0.095 (0.073, 0.117)	0.235 (0.189, 0.281)
Gestation (weeks)^c^	− 0.001 (− 0.002, 0.0001)	− 0.002 (− 0.003, − 0.0004)	− 0.003 (− 0.004, − 0.001)	0.00004 (− 0.001, 0.001)	− 0.0003 (− 0.001, 0.0003)	− 0.001 (− 0.002, 0.0004)
High immune						
Mean antibody level^b^	0.864 (0.67, 1.062)	1.076 (0.897, 1.264)	1.000 (0.893, 1.111)	0.603 (0.404, 0.808)	0.448 (0.283, 0.608)	0.634 (0.498, 0.766)
Gestation (weeks)^c^	− 0.0004 (− 0.006, 0.005)	0.0001 (− 0.005, 0.005)	0.001 (− 0.002, 0.005)	− 0.006 (− 0.012, − 0.001)	− 0.003 (− 0.008, 0.002)	0.002 (− 0.003, 0.006)

Posterior medians (95% credible interval) for the fixed effects, population average intercept and population average slope parameters

^a^Fixed effects interpretation: change in mean antibody levels per year increase in age and difference in mean antibody levels for primigravidae vs. multigravida (reference group), chloroquine vs. placebo (reference group) and history vs. no history of malaria (reference group)

^b^Population mean antibody levels for women of average age (25 years old), multigravida, in the placebo group and with no history of malaria

^c^Population average change in antibody levels for a one week increase in gestation

The posterior medians for the population average slope indicate that, on average, antibody responses to *Pf*AMA1 and *Pv*AMA1 decreased with gestational age in both the low and high immune groups. For *Pf*EBA175, *Pf*MSP2 and *Pf*VAR2CSA, the posterior medians for the population average slope indicated that, on average, antibody responses decreased with gestational age in the low immune group but increased with gestational age in the high immune group; these trends were reversed for antibody responses to *Pf*MSP3.

### Influence of specific antibodies on the classification into high and low immunity groups

The variable-specific entropy for the antibody responses to the six antigens are given in Table 3. Antibody responses to the antigen *Pf*MSP2 played a lesser role in cluster allocation (lowest entropy = 0.763), whereas the relatively high entropy values for *Pf*MSP3 (0.949) and *Pv*AMA1 (0.935) indicate that the allocation of women to the low or high immune group was predominantly determined by their antibody responses to these antigens. Therefore, additional analyses were performed to assess the importance of including both *Pf*MSP3 and *Pv*AMA1 antibodies in allocating a woman to the low or high immune group. These analyses found that at least one of the two antigens, *Pv*AMA1 or *Pf*MSP3, is necessary for correctly grouping pregnant women with low antibody profiles (Additional file 1: Supplementary methods, Sect. 7).

**Table 3:** Influence of specific antibodies on the classification into high and low immunity profiles

Antibody	Entropy value^a^
*Pf*MSP3	0.949
*Pv*AMA1	0.935
*Pf*VAR2CSA	0.873
*Pf*AMA1	0.844
*Pf*EBA175	0.817
*Pf*MSP2	0.763

^a^The entropy is a value between 0 and 1 and values closer to 1 indicate that an antibody highly contributes towards the classification of women to cluster 1 or 2. The influence on classification declines as the entropy value declines

### Antibodies that best identify malaria cases

Using all six antibody responses, the multivariate model performed well for the controls by classifying them into the low immune group (107 of 111 controls, 96.4%); whereas the cases were poorly identified by the antibody responses to the six antigens; 51 (39%) and 79 (61%) cases classified in high and low immune groups, respectively (9 unclassified—4 controls and 5 cases).

Analyses including each antigen separately (i.e. univariate linear mixed-effects modelling), found antibody responses to the antigen *Pf*AMA1 were best able to discriminate between cases and controls and classified 66% of controls in the low immune group (77 out of 115 controls) and 55% of cases in the high immune group (74 out of 135 cases) (Table 4). The model fit to antibody responses to *Pv*AMA1 was best at identifying the controls (95%, 109 of 115 controls), followed by *Pf*MSP3 (90%, 103 of 115 controls). The model fit to antibody responses to *Pf*EBA175 was best at identifying the majority of the cases (60%, 81 of 135 cases), followed by *Pf*AMA1 (55%, 74 of 135 cases). Although the model fits to antibody responses to *Pv*AMA1 and *Pf*EBA175 were best at identifying controls and cases, respectively, they were unable to accurately classify the pregnant women in the other exposure group (Table 4). Although antibodies are acquired to VAR2CSA from exposure to malaria infection in pregnancy, antibodies to VAR2CSA did not perform as well as several merozoite antigens.

**Table 4:** Performance of classifying controls to Cluster 1^a^ and cases to Cluster 2^b^ based on univariate analyses^c^

	Number of controls^d^ classified into Cluster 1 (%)	Number of cases^e^ classified into Cluster 2 (%)	Total women classified into the expected Cluster^f^ (%)
*Pf*AMA1	76 (66.1)	74 (54.8)	150 (60)
*Pf*VAR2CSA	86 (74.8)	57 (42.2)	143 (57.2)
*Pf*EBA175	61 (53.0)	81 (60.0)	142 (56.8)
*Pv*AMA1	109 (94.8)	28 (20.7)	137 (54.8)
*Pf*MSP3	103 (89.6)	33 (24.4)	136 (54.4)
*Pf*MSP2	63 (54.8)	67 (49.6)	130 (52)

^a^Cluster 1 (low immunity group)

^b^Cluster 2 (high immunity group)

^c^Antibodies are ordered from the highest to the lowest percentage of classifying pregnant women into the expected cluster

^d^Out of 115 controls

^e^Out of 135 cases

fComputed based on the decision made that Cluster 1 would represent controls and Cluster 2 would represent cases. The sum of controls classified into Cluster 1 and cases classified into Cluster 2 were then divided by the total pregnant women, i.e. 250 to obtain the percentage

Pairwise analyses of the antibody responses were further explored considering the top 3 antibodies which correctly identified > 55% of all women (cases and controls) into the expected cluster in the univariate analyses, i.e. *Pf*AMA1, *Pf*VAR2CSA and *Pf*EBA175 (Table 4). The results of selected pairwise multivariate analyses (Additional file 1: Tables S6, S7) indicate that antibody responses to both *Pf*AMA1 and *Pf*VAR2CSA were best at identifying the cases as most women were classified into the high immune group. This combination classified 63% (73 of 115) of the controls into the low immune group and 63% (85 of 135) of the cases into the high immune group. For classification of falciparum and vivax infections, antibody responses to *Pf*AMA1 and *Pf*VAR2CSA classified the majority of *P. falciparum* only (81% [43/53]) and both *P. falciparum* and *P. vivax* (77% [24/31]) infections as cases, but performed poorly at classifying *P. vivax* only infections as cases (27% [12/44]) (Additional file 1: Table S8).

## Discussion

Pregnant women may serve as an accessible sentinel population to estimate the burden of malaria [34] and potentially for sero-surveillance studies to detect infections. In this cohort, antibody responses to the malaria parasite were highly dynamic, varying greatly within and between women during pregnancy. Modelling the longitudinal antibody response to six different antigens simultaneously found that pregnant women were classified into two major clusters, high immune and low immune response groupings, where 96% of the women who did not have a malaria infection detected during pregnancy had a low immune response. Combined antibody responses to the antigens *Pf*AMA1 and *Pf*VAR2CSA were best for identifying exposure to malaria during pregnancy, suggesting that these two antibodies may be good candidates for sero-surveillance of malaria infections in pregnant women.

Antibodies specific for *Plasmodium* spp. blood-stage antigens, in particular *Pf*VAR2CSA, are an attractive candidate for sero-surveillance of malaria in pregnancy as they have been demonstrated as a biomarker of recent exposure to malaria during pregnancy (systematically reviewed in [17]) and to monitor changes in malaria transmission over time [35]. However, the antibody to *Pf*VAR2CSA when used on its own was not the best marker of malaria infection in pregnancy. A combination of antibodies to *Pf*VAR2CSA and the merozoite antigen *Pf*AMA1 was the most accurate indicator of exposure to malaria during pregnancy. Our results show that the overall immunity corresponding to the *P. falciparum* parasite is positively correlated with increasing age, potentially due to increased lifetime exposure to malaria [36, 37]. No strong correlation with gravidity was observed, even with the pregnancy-specific immunity (*Pf*VAR2CSA), which may reflect the specific epidemiology of malaria in pregnancy in low endemic settings [38]. Antibody responses to *Pf*MSP3 and *Pv*AMA1 were the least dynamic, influencing the classification of pregnant women into the low immunity cluster suggesting that they are less suitable serological markers of recent exposure to malaria during pregnancy. Conversely, these properties meant that they were the most useful antigens studied in classifying women as controls (i.e. parasite negative) so may have potential for use as part of a broader panel of antigens. Other biomarkers of exposure to *P. falciparum* and *P. vivax* infection have been identified in non-pregnant individuals and would be valuable to investigate in future studies [39, 40]. This is the first study to incorporate and combine pregnancy and non-pregnancy specific antibodies, so our findings provide a novel avenue for improved sero-surveillance studies to be validated in a range of transmission settings. A further consideration in the application of sero-surveillance is the longevity of antibodies. In this study population, we previously estimated that *Pf*VAR2CSA antibodies may have a very long half-life (30–50 years), whereas *Pf*AMA1 antibodies decay more rapidly (half-life 2–3 years) [18]. Inclusion of long lived antibody responses, such as *Pf*VAR2CSA, should be interpreted with caution as markers of malaria exposure.

Several methods have been developed to cluster individuals based on longitudinal data, but the majority can only cluster a single longitudinal trajectory from each individual [41–45] or assume that multiple longitudinal trajectories from a single individual are independent [46] and cannot allow samples taken at irregularly (or unevenly) spaced time points [47]. The multivariate mixture linear mixed model approach was selected because it can handle all the statistical complexities/issues posed by the antibody data in this study, i.e., it allows classification of irregularly sampled multivariate longitudinal antibody response data, it is not constrained to assume independence between the longitudinal antibody response profiles from an individual and covariates can be included on the mixture model parameters [31].

This study has several limitations to consider. First, the availability of only one antibody marker for *P. vivax*, *Pv*AMA1, limits what can be understood about the immune response of pregnant women exposed to vivax malaria. The antibody responses to *Pv*AMA1 remained relatively low compared to other antibodies (except for *Pf*MSP3, the only synthetic peptide antigen included in the study which may explain the lower immunogenicity of this *P. falciparum* antigen). Parasite density is believed to have a proportional relationship with antibody maintenance and boosting [48–50]. That these densities are lower in vivax infections compared to falciparum infections [51] could possibly explain the maintenance of *Pv*AMA1 antibody responses at low levels. Administration of chloroquine, preventing vivax episodes, could be a significant confounding factor in disrupting antibody responses to *Pv*AMA1 [24]. The association of chloroquine prophylaxis with *Pv*AMA1 responses was observed though to be in the counterintuitive direction, however, there were a greater proportion of women in the chloroquine prophylaxis arm with a history of *P. vivax* infection which increased marginally the *Pv*AMA1 response. This study also only assessed six antibody responses and one domain of VARCSA, therefore, there may be other antigens that are better candidates for sero-surveillance.

Another limitation of this study was that the detection of exposure to malaria was based on microscopy, thereby, failing to detect low density sub-microscopic infections [52]. However, women in the study were tested for malaria infections by microscopy at multiple timepoints during pregnancy, potentially reducing the likelihood of missing low density infections. Pregnant women who regularly access antenatal care comprise an attractive sentinel surveillance population to estimate malaria transmission. Indeed, malaria incidence data from pregnant women spanning 10 years at the very same antenatal clinics was shown to correlate with presentation of clinical malaria in children under 5 years of age [53]. However, further studies are required to determine the utility of this sentinel surveillance population to monitor malaria transmission and if this surveillance method is more sensitive than multiple blood smears to detect a low level parasitaemia. Additionally the generalizability of these antigen combinations to accurately detect exposure to submicroscopic infections needs to be determined in future studies given the high rates of submicroscopic infections in malaria endemic regions [54].

## Conclusions

This study demonstrates that the combination of anti-*Pf*AMA1 and anti-*Pf*VAR2CSA antibodies could be used as a potential biomarker of exposure to malaria during pregnancy. However, only 63% of the malaria infections during pregnancy were detected, therefore further investigation of other antibody markers is warranted. This combined antibody diagnostic tool may facilitate the detection of microscopic infections as an alternative to standard diagnostic methods, particularly in low transmission settings. This proposed malaria sero-surveillance tool may also enhance malaria control and progress efforts to eliminate malaria.

## Supplementary Information

**Additional file 1**: Supplementary methods. **Table S1**. Number and species of infections. **Table S2**. Penalized expected deviance (PED) values of the models with mixture distributions ranging from *K* = 1, 2, 3, 4 clusters. **Table S3**. Characteristics of the pregnant women by low and high immune antibody response profile clusters. **Table S4**. Assessment of the importance of combining *Pv*AMA1 and *Pf*MSP3, respectively with other *P. falciparum* antibodies. **Table S5**. The importance of including at least *Pf*MSP3 or *Pv*AMA1 with other *Plasmodium falciparum* antibodies investigated. **Table S6**. Classification of pregnant women based on the joint antibody responses of *Pf*AMA1 and *Pf*VAR2CSA. **Table S7**. Classification of pregnant women based on the joint antibody responses of *Pf*AMA1 and *Pf*EBA175, and *Pf*EBA175 and *Pf*VAR2CSA, respectively. **Table S8**. Classification of cases by the type of infection, based on antibody responses to *Pf*AMA1 and *Pf*VAR2CSA. **Figure S1**. Longitudinal antibody levels against six antigens for all women free from malaria (controls) by the serostatus to schizont extract. **Figure S2**. Longitudinal antibody levels against six antigens for all women by gravidity (primigravidae versus multigravidae). **Figure S3**. Posterior cumulative distribution functions of the observed data deviances for models with *K* = 1, 2, 3, 4 clusters.

## Data Availability

The datasets used and/or analysed during the current study are available from the corresponding author and study investigators on reasonable request.

## Abbreviations

AMA1: Apical membrane antigen 1
ANCs: Antenatal clinics
CQ: Chloroquine
CrI: Credible interval
CI: Confidence interval
CSA: Chondroitin sulphate A
EBA: Erythrocyte binding antigen
IEs: Infected erythrocytes
IQR: Inter-quartile range
LOESS: Locally estimated scatterplot smoothing
MCMC: Markov chain Monte Carlo
MSP: Merozoite surface protein
SMRU: Shoklo Malaria Research Unit
VAR2CSA: Variant surface antigen 2-CSA
