# Supplementary material for: Quantification of the dynamics of antibody response to malaria to inform sero-surveillance in pregnant women
Source: Malar J. 2022 Mar 5;21:75. doi: 10.1186/s12936-022-04111-y (PMC8897879; doi:10.1186/s12936-022-04111-y)
Supplement: Supplementary file 1 — Additional file 1: Supplementary methods. Table S1. Number and species of infections. Table S2. Penalized expected deviance (PED) values of the models with mixture distributions ranging from K = 1, 2, 3, 4 clusters. Table S3. Characteristics of the pregnant women by low and high immune antibody response profile clusters. Table S4. Assessment of the importance of combining PvAMA1 and PfMSP3, respectively with other P. falciparum antibodies. Table S5. The importance of including at least PfMSP3 or PvAMA1 with other Plasmodium falciparum antibodies investigated. Table S6. Classification of pregnant women based on the joint antibody responses of PfAMA1 and PfVAR2CSA. Table S7. Classification of pregnant women based on the joint antibody responses of PfAMA1 and PfEBA175, and PfEBA175 and PfVAR2CSA, respectively. Table S8. Classification of cases by the type of infection, based on antibody responses to PfAMA1 and PfVAR2CSA. Figure S1. Longitudinal antibody levels against six antigens for all women free from malaria (controls) by the serostatus to schizont extract. Figure S2. Longitudinal antibody levels against six antigens for all women by gravidity (primigravidae versus multigravidae). Figure S3. Posterior cumulative distribution functions of the observed data deviances for models with K = 1, 2, 3, 4 clusters. [file 12936_2022_4111_MOESM1_ESM.docx]

**Supplementary material**

**Quantification of the dynamics of antibody response to malaria in pregnant women**

**Contents:**

Supplementary methods

Antibody determination

Statistical Analyses

1. Linear mixed model with a normal mixture for a single outcome
2. Multivariate linear mixed model with normal mixture in the random effects distribution
3. Specification of model parameters and parameter estimation
4. Clustering procedure
5. Selection of the optimal number of mixture components
6. Computation of entropy value specific to each antibody
7. Importance of including *Pf*MSP3 and *Pv*AMA1

Supplementary Table 1: Number and species of infections

Supplementary Table 2. Penalized expected deviance (PED) values of the models with mixture distributions ranging from *K* = 1, 2, 3, 4 clusters.

Supplementary Table 3. Characteristics of the pregnant women by low and high immune antibody response profile clusters.

Supplementary Table 4. Assessment of the importance of combining *Pv*AMA1 and *Pf*MSP3, respectively with other *P. falciparum* antibodies.

Supplementary Table 5. The importance of including at least *Pf*MSP3 or *Pv*AMA1 with other *Plasmodium falciparum* antibodies investigated.

Supplementary Table 6. Classification of pregnant women based on the joint antibody responses of *Pf*AMA1 and *Pf*VAR2CSA.

Supplementary Table 7. Classification of pregnant women based on the joint antibody responses of *Pf*AMA1 and *Pf*EBA175 and *Pf*EBA175 and *Pf*VAR2CSA, respectively.

Supplementary Table 8. Classification of cases by the type of infection, based on antibody responses to *Pf*AMA1 and *Pf*VAR2CSA.

Supplementary Figure 1. Longitudinal antibody levels against six antigens for all women free from malaria (controls) by the serostatus to schizont extract.

Supplementary Figure 2. Longitudinal antibody levels against six antigens for all women by gravidity (primigravidae versus multigravidae).

Supplementary Figure 3. Posterior cumulative distribution functions of the observed data deviances for models with *K* = 1, 2, 3, 4 clusters.

Supplementary Material References

**Supplementary Methods**

**Antibody determination**

ELISAs were performed using established methods (2). Ninety-six well Maxisorp plates [Nunc, Roskilde, Denmark] were coated with schizont lysate at a concentration of 1:500 in PBS and were incubated overnight at 4°C. Plates were washed then blocked with 10% skim milk powder in PBS-tween 0.05% for 2 hours at 37°C. After washing, serum samples, at 1:2000 in 5% skim milk powder in PBS-tween 0.05%, were incubated for 2 hours. Plates were washed and incubated for 1 hour with HRP-conjugated sheep anti-human IgG (Millipore, North Ryde, Australia) at 1:5000 in 5% skim milk powder in PBS-tween 0.05%. After washing, colour was developed by adding ABTS [2,2’-azinobis(3-ethylbenzthiazolinesulfonic acid)] substrate (Sigma-Aldrich, Castle Hill, Australia) and the reaction was stopped using 1% sodium dodecyl sulfate (SDS). The optical density (OD) of each sample was measured at 405nm. All washes were carried out using PBS-tween 0.05% and all incubations occurred at room temperature unless otherwise stated. Samples were tested in duplicate (100µl/well). Standardization of the plates was achieved using positive-control plasma pools on each plate. Background (determined from the wells with no serum) was subtracted from each duplicate and the mean of each sample was calculated. A cut-off threshold for sero-positivity was determined using the mean plus 3 standard deviations of eight negative controls (non-exposed Melbourne donors).

**Statistical Analyses**

**Notation**

Data from a total of 250 pregnant women were analysed. The follow-up times, $\boldsymbol{t}_{i}= \left( t_{i,1}, \ldots, t_{i,n_{i}} \right)^{T},$correspond to the $i^{th}$ woman with $n_{i}$ ANC visits from enrolment to delivery; the number of ANC visits and the visit times can vary between the women. Let $\boldsymbol{Y}_{\boldsymbol{i,r}}= \left( Y_{i,r,1}, \ldots, Y_{i,{r,n}_{i}} \right)^{T}$, *r* = 1, …, 6, represent the vector of the longitudinal measurements of the $i^{th}$ woman on the$r^{th}$ antibody and $\boldsymbol{Y}_{\boldsymbol{i}} = \left( {\boldsymbol{Y}_{\boldsymbol{i,1}}}^{T}, \ldots, {\boldsymbol{Y}_{\boldsymbol{i,6}}}^{T} \right)^{T}$denote the complete longitudinal antibody data for the$i^{th}$ woman.

**1. Multivariate linear mixed model (MLMM)** **with a normal mixture for a single outcome**

Initially, a standard linear mixed model was fitted to each of the six antibody responses with the following covariates as fixed effects (***X***): age (a continuous variable, years), primigravidae (a dichotomous variable assigned 1 if primigravidae), treatment arm (a dichotomous variable assigned 1 if given chloroquine (CQ) as prophylaxis at enrollment) and having a history of malaria prior to enrollment (a dichotomous variable assigned 1 if exposed to malaria at least once prior to enrollment). Antibody measures at conception (intercept) and the rate of change of the antibody response over gestational time (in weeks) were included as random effects to allow variation across women in each antibody profile. Therefore, the linear mixed effects model fitted on each antibody is defined by:

$\boldsymbol{Y}_{\boldsymbol{i,r}}\boldsymbol{=}\boldsymbol{X}_{\boldsymbol{i,r}}\boldsymbol{\alpha}_{\boldsymbol{r}}\boldsymbol{+}\boldsymbol{Z}_{\boldsymbol{i,r}}\boldsymbol{b}_{\boldsymbol{i,r}}\boldsymbol{+}\boldsymbol{\varepsilon}_{\boldsymbol{i,r}}, i=1, \ldots, 250, r=1, \ldots, 6, \left( 1 \right)$

where,

$\boldsymbol{X}_{\boldsymbol{i}\boldsymbol{,}\boldsymbol{r}}$: $n_{i,r}$× 4 covariate matrix for fixed effects

$\boldsymbol{Z}_{\boldsymbol{i}\boldsymbol{,}\boldsymbol{r}}$: $n_{i,r}$× 2 covariate matrix for random effects

$\boldsymbol{\alpha}_{\boldsymbol{r}}$ = ${(\alpha_{r,age}, \alpha_{r,primigravidae}, \alpha_{r,CQ}, \alpha_{r,history} )}^{T}$ : vector of fixed effects coefficients for *r^th^* antibody

$\boldsymbol{b}_{\boldsymbol{i}\boldsymbol{,}\boldsymbol{r}}$ = ${(b_{i,r,intercept}, b_{i,r,slope} )}^{T}$ : vector of random effects coefficients for *r^th^* antibody specific to the $i^{th}$ woman

$\boldsymbol{\varepsilon}_{\boldsymbol{i}\boldsymbol{,}\boldsymbol{r}}$ = ${(\boldsymbol{\varepsilon}_{i,r,1}, \ldots, \boldsymbol{\varepsilon}_{i,r,n_{i,r}} )}^{T}$ : vector of random errors for the measurements of antibody *r* of the $i^{th}$ woman

The errors were assumed to be mutually independent and normally distributed, with a specific variance for each antibody ($\boldsymbol{\phi}_{r})$, defined by:

$$\boldsymbol{\varepsilon}_{\boldsymbol{i}\boldsymbol{,}\boldsymbol{r}} \sim N\left( 0,\boldsymbol{\phi}_{r} \right)\boldsymbol{.}$$

**2. Multivariate linear mixed model for multiple antibodies with normal mixture in the random effects distribution**

**Model specification:**

In mixAK package (3), the clustering is performed by fitting a multivariate mixture linear mixed model (MMLMM) to the data of all six antibody measures simultaneously:

$$\boldsymbol{Y}_{\boldsymbol{i}}\boldsymbol{=}\boldsymbol{X}_{\boldsymbol{i}}\boldsymbol{\alpha+}\boldsymbol{Z}_{\boldsymbol{i}}\boldsymbol{b}_{\boldsymbol{i}}\boldsymbol{+}\boldsymbol{\varepsilon}_{\boldsymbol{i}}, i=1,\ldots, 250, (2)$$

where,

$\boldsymbol{X}_{\boldsymbol{i}}$: $n_{i}$ × 24 block-diagonal matrix with $X_{i,1}, \ldots, X_{i,6}$ matrices on the diagonal

$\boldsymbol{Z}_{\boldsymbol{i}}$: $n_{i}$ × 12 block-diagonal matrix with $Z_{i,1}, \ldots, Z_{i,6}$ matrices on the diagonal

$\boldsymbol{\alpha}$ = ${(\alpha_{1}^{T},\ldots,\alpha_{6}^{T} )}^{T}$ : vector of fixed effects for all antibody responses

$\boldsymbol{b}_{\boldsymbol{i}}$= $\left( {b_{i,1}}^{T}, \ldots, {b_{i,6}}^{T} \right)^{T}$: The vector of random effects of the $i^{th}$ woman on all antibody responses

$\boldsymbol{\varepsilon}_{\boldsymbol{i}}$ = $\left( {\varepsilon_{i,1}}^{T}, \ldots, {\varepsilon_{i,6}}^{T} \right)^{T}$: The vector of errors of the $i^{th}$ woman on all antibody responses

**Distribution of random effects**

Correlation between antibody responses is introduced by defining a joint distribution for $\boldsymbol{b}_{\boldsymbol{i}}$=$\left( {b_{i,1}}^{T}, \ldots, {b_{i,6}}^{T} \right)^{T}$, given by:

$b_{i}\sim N\left( \boldsymbol{\beta},\boldsymbol{D} \right),$

where, $\boldsymbol{\beta}$ is the mean and ***D*** is the covariance matrix (capturing the correlation between antibody responses) of the overall distribution of the random effects $b_{i}$, $i=1, \ldots, 250$.

For determining clusters of antibody response profiles, the normality assumption of the random effects is replaced by a mixture of $K$ normal distributions:

$\boldsymbol{b}_{\boldsymbol{i}}\sim\sum_{k=1}^{K} w_{k}N\left( \boldsymbol{\mu}_{\boldsymbol{k}},\boldsymbol{D}_{\boldsymbol{k}} \right)$,

where the mixture distribution is a multivariate normal distribution and comprises of a unique unknown mean $\boldsymbol{\mu}_{\boldsymbol{k}}$ and unknown covariance matrix $\boldsymbol{D}_{\boldsymbol{k}}$, $k=1,\ldots, K$. The vector, $\boldsymbol{w}=\left( w_{1}, \ldots, w_{K} \right)^{T}$ ($w_{i}>0$ and $\sum_{i=1}^{K} w_{i}=1$) contains the proportion of women being classified into each cluster. The details of classifying the pregnant women into *K* clusters of longitudinal antibody response profiles is provided in Supplementary Methods 4.

**3. Specification and estimation of the model parameters**

Some of the parameters are common across clusters and the others are specific to each cluster, as explained below.

**Model parameters common to all clusters**

The model parameters common to all clusters include the fixed effects (i.e., the parameters for age, primigravidae, treatment arm and history of malaria prior to enrollment) and the error variances, i.e.,

$$\boldsymbol{\psi=}\left( {\boldsymbol{\alpha}_{\boldsymbol{1}}}^{\boldsymbol{T}}\boldsymbol{, \ldots,}{\boldsymbol{\alpha}_{\boldsymbol{6}}}^{\boldsymbol{T}}\boldsymbol{,}\boldsymbol{\phi}_{\boldsymbol{1}}\boldsymbol{, \ldots,}\boldsymbol{\phi}_{\boldsymbol{6}} \right)^{\boldsymbol{T}}\boldsymbol{.}$$

Since, four fixed effects and an error variance were introduced for each antibody response, $\boldsymbol{\psi}$ is comprised of 24 fixed effects and 6 residual error variances.

**Model parameters specific to each cluster**

The parameters corresponding to the distributions of the random effects were cluster specific, i.e.,

$\boldsymbol{\theta=}\left( \boldsymbol{w}^{T}\boldsymbol{,}{\boldsymbol{\mu}_{\boldsymbol{1}}}^{T},\ldots,{\boldsymbol{\mu}_{\boldsymbol{K}}}^{T},\mathbf{vec}\left( \mathbb{D}_{1} \right),\ldots,\mathbf{vec}\left( \mathbb{D}_{k} \right) \right)^{\boldsymbol{T}}$**,**

where **vec**$\left( \mathbb{D}_{k} \right)$ represents the vector of elements in the lower triangle of the matrix $\boldsymbol{D}_{\boldsymbol{k}}$.

**Parameter estimation**

A Bayesian approach with Markov chain Monte Carlo (MCMC) simulation method was incorporated in the process of estimating unknown model parameters $(\boldsymbol{\psi,\theta})$.

Initially, prior distributions of the model parameters were specified. Independence between the mixture parameters $\boldsymbol{\theta}$ and the parameters $\boldsymbol{\psi}$ (common to all clusters) was assumed, hence their prior joint distribution is defined as $p\left( \boldsymbol{\psi,\theta} \right)$= $p(\boldsymbol{\psi})p(\boldsymbol{\theta}$). For $p(\boldsymbol{\theta}$), the multivariate version of the priors proposed by Richardson and Green (1997) (4), which is defined for univariate normal mixtures (e.g., $\boldsymbol{D}_{\boldsymbol{k}}^{\boldsymbol{-1}}$ follows a gamma distribution), was selected, and for $p\left( \boldsymbol{\psi} \right)$, we adopted priors used typically for the fixed-effects and residual error in univariate linear mixed-effect modelling (e.g., $\boldsymbol{\alpha}$ is assumed to be normally distributed, see Fong, Rue and Wakeﬁeld (2010) (5)). Hyperparameters were then introduced so that the prior distribution of $p(\boldsymbol{\psi,\theta})$ will be weakly informative (refer to Appendix A of the Supplementary Material of Komarek and Komarkova (2013) (6) for a detailed explanation of specification of the hyperparameters).

Next, the likelihood of the MMLMM is formed as below:

$L\left( \boldsymbol{\psi,\theta} \right)=p\left( \boldsymbol{y} | \boldsymbol{\psi,\theta} \right)= \prod_{i=1}^{250} \left( \sum_{k=1}^{K} w_{k}L_{i,k}\left( \boldsymbol{\psi,\theta} \right) \right),$

where $L_{i,k}\left( \boldsymbol{\psi,\theta} \right)$ is the contribution of the$i^{th}$woman to the likelihood assuming that the woman was classified into the $k^{th}$ mixture component, defined by

$L_{i,k}\left( \boldsymbol{\psi}\boldsymbol{,}\boldsymbol{\theta} \right)= \int\left\{ \prod_{r=1}^{6} \prod_{j=1}^{n_{i}} p\left( y_{i,r,j}|\boldsymbol{\phi}_{\boldsymbol{r}}\boldsymbol{,}\boldsymbol{\alpha}_{\boldsymbol{r}\boldsymbol{,}}\boldsymbol{b}_{\boldsymbol{i}\boldsymbol{,}\boldsymbol{r}} \right) \right\}p\left( b_{i} | \boldsymbol{\theta},U_{i}= k \right){db}_{i}$ $i=1,\ldots,250 k=1,.., K.$

Subsequently, the MCMC method was used to derive *M* number of samples of model parameters, $\boldsymbol{S}_{\boldsymbol{M}}= \left\{ \left( \boldsymbol{\psi}^{\boldsymbol{(m)}},\boldsymbol{\theta}^{\boldsymbol{(m)}} \right):m=1, \ldots,M \right\},$ from the joint posterior distribution, $p\left( \boldsymbol{\psi,\theta}|y \right)\propto L\left( \boldsymbol{\psi,\theta} \right)p\left( \boldsymbol{\psi,\theta} \right).$

Of note, the problem of completely different sets of parameters resulting in similar mixture distributions was addressed by generating posterior parameter estimates using the relabeling algorithm of Stephens (2000) (7).

The parameters of the fitted MMLMM with *K* mixture components were estimated using 50,000 posterior MCMC samples generated from the joint posterior distribution under two parallel chains, following 500 burn-in and 1:50 thinning. Of note, due to computational time, the selection of the number of mixture components was based on a sample of 10,000 iterations obtained after a burning period of 500 and thinning of 1:10.

**4. Clustering procedure**

Classification of pregnant women into *K* clusters was based on the posterior probabilities of belonging to each *K* cluster generated for each woman, given by:

$p_{i,k}= p_{i,k} \left( \boldsymbol{\theta} \right)= P\left( U_{i}=k|\boldsymbol{Y}_{\boldsymbol{i}}\boldsymbol{=}\boldsymbol{y}_{\boldsymbol{i}}; \boldsymbol{\theta} \right)= \frac{w_{k}f_{i,k}\left( \boldsymbol{Y}_{\boldsymbol{i}};\boldsymbol{\theta}_{\boldsymbol{k}}\boldsymbol{, \psi} \right)}{f_{i}\left( \boldsymbol{Y}_{\boldsymbol{i}},\boldsymbol{\theta} \right)}, i=1,\ldots,250;k= 1, \ldots. , K$,

where $f_{i,k}\left( \boldsymbol{Y}_{\boldsymbol{i}};\boldsymbol{\theta}_{\boldsymbol{k}}\boldsymbol{,\psi} \right)$ is the conditional density of $\boldsymbol{Y}_{\boldsymbol{i}}$ given $U_{i}=k$ ($U_{i}$ ϵ $\left\{ 1, \ldots, K \right\}$ denotes the assignment of the $i^{th}$ woman to a cluster). The values of $p_{i,k}$ were estimated from the 50,000 posterior samples of the model parameters.

At each iteration of the MCMC sampling method, probabilities of falling into each cluster was estimated for the women. Next, the posterior median, posterior mean and the 95% credible interval of the resultant 50,000 chain of the probabilities were calculated for each woman. The classification was then performed based on these summary measures under two steps, detailed below.

**Step 1:**

Each pregnant woman was assigned to the cluster with the highest mean/median of the estimated probabilities, $p_{i,k}$. The classification is performed using both estimates of mean and median and for our antibody data both the mean and median classified the same pregnant women into the two clusters.

**Step 2:**

Once a cluster is nominated using Step 1, the woman will remain in that cluster if the lower limit of the 95% credible interval computed for the probabilities of that cluster exceeds 0.5, otherwise she is considered unclassified.

**5. Selection of the optimal number of mixture components**

The optimal number of mixture distributions that best explains the overall distribution of the random effects was determined using two approaches:

1. **Penalized expected deviance (PED)**

Penalized expected deviance (PED), which is a goodness of fit measure, was introduced by Plummer (2008) (8) to use in mixture models, as follows:

$$PED=E\left\{ D\left( \boldsymbol{\psi},\boldsymbol{\theta} \right)|\boldsymbol{Y=y} \right\}+ P_{opt},$$

where, the first term is the expected deviance, given ***Y = y***, which is computed by averaging the data deviance $D\left( \boldsymbol{\psi},\boldsymbol{\theta} \right)$ observed at each MCMC simulation. The second term, $P_{opt}$, is a penalty term called optimism, introduced to account for the adequacy of the model, and captures the amount by which the model adequacy is overstated by each woman ($\boldsymbol{Y}_{\boldsymbol{i}}$) (for further details see (8)). In this approach, the optimal number of clusters is the one that results in the lowest PED.

1. **Plot of posterior distribution of the deviances**

According to Aitkin (2010) (9), the optimal number of clusters can be determined by comparing the distribution of the cumulative posterior distributions (CDFs) of the deviances of the MCMC samples, as opposed to the PED approach which bases the clustering on a single estimated value. In this approach, the optimal number of clusters is the one which maintains the deviance at the lowest level throughout the simulation.

**6.** **Computing entropy values for antibodies**

The importance level of each antibody in clustering the pregnant women was determined by computing univariate entropy value $\left( E_{r} \right)$ for each antibody (10) as follows:

$E_{r}=1+\frac{1}{N log(K)}\left( \sum_{i=1}^{N} \sum_{k=1}^{K} P\left( U_{i}=k\left| \boldsymbol{Y}_{\boldsymbol{ir}} \right. \right)\log\left( P\left( U_{i}=k\left| \boldsymbol{Y}_{\boldsymbol{ir}} \right. \right) \right) \right)$, $r=1,\ldots, 6,$

where $N$ is the sample size (here, $N$ = 250 pregnant women), $K$ is the number of classes ($K$ = 2 for the antibody response profiles) and $\boldsymbol{Y}_{\boldsymbol{i}}$ represents the longitudinal trajectories of all six antibody responses corresponding to the $i^{th}$ woman, upon which the classification was made.

The probability of classifying the $i^{th}$ woman to the $k^{th}$ cluster using only the $r^{th}$ antibody $\left( P\left( U_{i}=k\left| \boldsymbol{Y}_{\boldsymbol{ir}} \right. \right) \right)$, where $\boldsymbol{Y}_{\boldsymbol{ir}}$ is the subset of $\boldsymbol{Y}_{\boldsymbol{i}}$ corresponding to the $r^{th}$ antibody, was computed by (6):

$P \left( U_{i}=k\left| \boldsymbol{Y}_{\boldsymbol{ir}} \right. \right)= \frac{w_{k}N\left( \boldsymbol{b}_{\boldsymbol{ir}}^{\boldsymbol{*}}\left| \boldsymbol{\mu}_{\boldsymbol{kr}}^{\boldsymbol{*}} \right.,\boldsymbol{D}_{\boldsymbol{kr}}^{\boldsymbol{*}} \right)}{\sum_{l=1}^{K} w_{l} N\left( \boldsymbol{b}_{\boldsymbol{ir}}^{\boldsymbol{*}}\left| \boldsymbol{\mu}_{\boldsymbol{lr}}^{\boldsymbol{*}} \right.,\boldsymbol{D}_{\boldsymbol{lr}}^{\boldsymbol{*}} \right)}$ $k=1,\ldots., K$,

where $\boldsymbol{b}_{\boldsymbol{i}}^{\boldsymbol{*}}$ is a standardised version of $\boldsymbol{b}_{\boldsymbol{i}}$ used for improving the mixing and numerical stability of the MCMC algorithm (6).

Of note, the entropy is a value between 0 and 1 and an antibody with an entropy value closer to 1 indicates that the separation of pregnant women is highly influenced by the specific antibody.

**7. Importance of including *Pf*MSP3 and *Pv*AMA1**

According to the variable-specific entropy values in Table 3, antibody responses to *Pf*MSP3 and *Pv*AMA1 predominantly determined whether a woman was allocated to the low or high immune group. The biological mechanisms of these antigens are quite different from the other antibodies (i.e. *Pv*AMA1 is a *P. vivax* antigen while *Pf*MSP3 does not connect directly to the merozoite surface as the four other *P. falciparum* proteins). Hence, how important including antibody responses to these two antigens are in allocating a woman to the low or high immune group was further assessed by repeating the clustering analysis with and without these two antibodies.

The clusters from analyses excluding either *Pv*AMA1 or *Pf*MSP3 accurately reproduced the clusters from the analysis including all six antigens (Supplementary Table 2). Of the 186 and 55 women allocated to clusters 1 (low immune) and 2 (high immune), respectively, in the analysis including all antigens, 96% (178/186) and 87% (48/55) of women were allocated to clusters 1 and 2 in the analysis excluding *Pv*AMA1. Similar percentages were observed for the analysis excluding *Pf*MSP3.

A cross tabulation of the clusters from the analysis including all antigens with those from the analysis excluding both *Pv*AMA1 and *Pf*MSP3 is presented in Supplementary Table 3. The four *P. falciparum* antibodies remaining in the analysis after excluding *Pv*AMA1 and *Pf*MSP3 were able to accurately reproduce the cluster 2 (high immune) allocation from the analysis including all antigens. Of the 55 women classified into cluster 2 in the analysis including all antigens, 95% (53/55) were also classified into cluster 2 in the analysis excluding *Pv*AMA1 and *Pf*MSP3. The analysis excluding both *Pv*AMA1 and *Pf*MSP3 was not able to accurately reproduce the cluster 1 (low immune) allocation from the analysis including all antigens. Of the 186 women classified into cluster 1 in the analysis including all antigens, only 47% (87/186) were classified into cluster 1 in the analysis excluding *Pv*AMA1 and *Pf*MSP3.

At least one of the two antigens *Pv*AMA1 or *Pf*MSP3 (*Pf*MSP3 is preferred as it has the highest variable-specific entropy and is a *P. falciparum* antigen) should be considered in cluster analyses to ensure pregnant women who tend to have low antibody responses over gestational age are classified into cluster 1 (the low immune cluster identified in the analysis of all antigens).

**Supplementary Table 1: Number and species of infections**

| Number of infections^1^ per woman | Type of infection(s)^1^ recorded during pregnancy | Count |
| --- | --- | --- |
| 1  (n = 69) | *P. falciparum* | 38 |
|  | *P. vivax* | 31 |
| 2  (n = 30) | Both *P. falciparum* and *P. vivax* | 9 |
|  | *P. falciparum* | 14 |
|  | *P. vivax* | 6 |
|  | *P. vivax* and *P. malariae* infection | 1 |
| 3  (n = 36) | Both *P. falciparum* and *P. vivax* | 22 |
|  | *P. falciparum* | 1 |
|  | *P. falciparum* and *P. malariae* infection | 1 |
|  | *P. falciparum*, *P. vivax* and mixed infection | 4 |
|  | *P. falciparum*, *P. vivax* and *P. malariae* | 1 |
|  | *P. vivax* | 7 |

^1^Cannot distinguish between new and recrudescent infections.

**Supplementary Table 2. Penalized Expected Deviance (PED) estimated for the models with the number of mixture distributions (i.e. clusters) ranging from one to four.**

| $K$ | Estimate of average deviance | $P_{opt}$ | Penalised Expected Deviance (PED) |
| --- | --- | --- | --- |
| 1 | -6183.12 | 220.8 | -5962.31 |
| 2 | -7442.12 | 473.06 | -6969.06 |
| 3 | 1374.62 | 2005.26 | 3379.87 |
| 4 | 1026.26 | 2570.99 | 3597.25 |

PED – sum of average deviance and $P_{opt}$

$K$ = 2 provided the minimum PED value, i.e., a multivariate linear mixed model with a mixture of two random effects distributions best explains variation in the antibody profiles of the pregnant women. Hence, it was determined to classify the pregnant women into two groups.

**Supplementary Table 3. Characteristics of the pregnant women by low and high immune antibody response profile clusters.^a^**

| **Characteristic** | **Cluster 1**  **Low immune group**  **(n=186)** | **Cluster 2**  **High immune group**  **(n=55)** |
| --- | --- | --- |
| **Exposed to malaria during pregnancy** |  |  |
| Case, n (%) | 79 (42.5) | 51 (92.7) |
| *P. falciparum* only, n (%)  *P. vivax* only, n (%)  Both *P. falciparum* and *P. vivax,* n (%)  *P. falciparum, P. vivax* and mixed infection,  n (%)  Other three infections^b^, n (%) | 23 (29.1)  38 (48.1)  16 (20.3)  2 (2.5)  0 (0) | 28 (54.9)  5 (9.8)  13 (25.5)  2 (3.9)  3 (5.9) |
|  |  |  |
| Control, n (%) | 107 (57.5) | 4 (7.3) |
| Age (years), median (IQR) | 25 (21, 31) | 24 (20, 30.5) |
| **Gravidity, median (IQR)** | 3 (2, 5) | 3 (2, 5.5) |
| Primigravida, n (%) | 33 (17.7) | 13 (23.6) |
| Multigravida, n (%) | 153 (82.3) | 42 (76.4) |
| Parity, median (IQR) | 2 (1, 3) | 1 (0, 3.5) |
| Haematocrit (%), median (IQR) | 33.8 (31.5, 36) | 31.5 (27.6, 33.8) |
| Anaemia, n (%) | 23 (12.4) | 19 (34.5) |
| Residence in refugee camp, n (%) | 146 (78.5) | 23 (41.8) |
| Receiving chloroquine prophylaxis, n (%) | 85 (45.5) | 24 (42.9) |
| Estimated Gestational Age (weeks), median (IQR) | 9.3 (7.3, 12.3) | 10.9 (7.8, 16.8) |
| **Trimester** |  |  |
| 1 (<14wks), n (%) | 157 (84.4) | 36 (65.5) |
| 2 (14 to <28wks), n (%) | 28 (15.1) | 18 (32.7) |
| 3 (28 wks or more), n (%) | 1 (0.5) | 1 (1.8) |
| ***Plasmodium* spp. before enrolment^c^, n (%)** | 89 (47.8) | 26 (47.3) |
| Infected with *P. falciparum*^d^, n (%) | 59 (32.1) | 21 (38.9) |
| Infected with *P. vivax*^d^, n (%) | 39 (21.2) | 6 (11.1) |
| Follow up (weeks), median (range) | 30.8 (26.6, 32.4) | 26.3 (19, 31.1) |

^a^9 women who were not classified into any cluster were excluded (5 cases and 4 controls).

^b^3 other infections include *P. falciparum* and *P. malariae* infection, *P. vivax* and *P. malariae* infection and *P. falciparum*, *P. vivax* and *P. malariae* infection with one pregnant woman in each type of infection.

^c^ Any microscopically confirmed *Plasmodium* infection documented at SMRU before enrolment into the study.

^d^ Includes women that have a history of both *P. falciparum* and *P. vivax* infections. Hence, the summation of *P. falciparum*^d^ and *P. vivax*^d^ women does not add up to *Plasmodium* spp. before enrolment^c^.

**Supplementary Table 4. Assessment of the importance of combining *Pv*AMA1 and *Pf*MSP3, respectively with other *P. falciparum* antibodies.**

|  | Cluster groupings considering  all antibodies  without *Pv*AMA1 | | | Cluster groupings considering  all antibodies  without *Pf*MSP3 | | |  |
| --- | --- | --- | --- | --- | --- | --- | --- |
| Cluster groupings considering all antibodies | Cluster 1 | Cluster 2 | Un-classified | Cluster 1 | Cluster 2 | Un-classified | Total |
| Cluster 1 | 178 | 0 | 8 | 173 | 1 | 12 | 186 |
| Cluster 2 | 3 | 48 | 4 | 3 | 49 | 3 | 55 |
| Un-  classified | 2 | 4 | 3 | 3 | 3 | 3 | 9 |
| Total | 183 | 52 | 15 | 179 | 53 | 18 | 250 |

**Supplementary Table 5. The importance of including at least *Pf*MSP3 or *Pv*AMA1 with other *Plasmodium falciparum* antibodies investigated.**

|  |  | Cluster groupings excluding both *Pv*AMA1 and *Pf*MSP3 | | |  |
| --- | --- | --- | --- | --- | --- |
|  |  | Cluster 1 | Cluster 2 | Unclassified | Total |
| Cluster groupings obtained considering all antibodies | Cluster 1 | 87 | 79 | 20 | 186 |
|  | Cluster 2 | 2 | 53 | 0 | 55 |
|  | Unclassified | 1 | 8 | 0 | 9 |
|  | Total | 90 | 140 | 20 | 250 |

| Exposure | Cluster 1 | Cluster 2 | Unclassified | Total |
| --- | --- | --- | --- | --- |
| Case | 41 | 85 | 09 | 135 |
| Control | 73 | 26 | 16 | 115 |
| Total | 114 | 111 | 25 | 250 |

**Supplementary Table 6. Classification of pregnant women based on the joint antibody responses of *Pf*AMA1 and *Pf*VAR2CSA.**

**Supplementary Table 7. Classification of pregnant women based on the joint antibody responses of *Pf*AMA1 and *Pf*EBA175 and *Pf*EBA175 and *Pf*VAR2CSA, respectively.**

| Exposure | Cluster grouping considering *Pf*AMA1 and *Pf*EBA175 | | | Cluster grouping considering *Pf*EBA175 and *Pf*VAR2CSA | | | Total |
| --- | --- | --- | --- | --- | --- | --- | --- |
|  | Cluster 1 | Cluster 2 | Unclassified | Cluster 1 | Cluster 2 | Unclassified |  |
| Case | 42 | 84 | 9 | 46 | 84 | 5 | 135 |
| Control | 52 | 51 | 12 | 61 | 43 | 11 | 115 |
| Total | 94 | 135 | 21 | 107 | 127 | 16 | 250 |

Both these combinations identify cases almost similarly to the bivariate combination *Pf*AMA1 and *Pf*VAR2CSA but poorly identify the controls and classify them into Cluster 1.

**Supplementary Table 8: Classification of cases by the type of infection, based on antibody responses to *Pf*AMA1 and *Pf*VAR2CSA**

| Type of infection(s) recorded during pregnancy | Cluster 1 | Cluster 2 | Unclassified | Total |
| --- | --- | --- | --- | --- |
| *P. falciparum* only | 5 | 43 | 5 | 53 |
| Both *P. falciparum* and *P. vivax* | 6 | 24 | 1 | 31 |
| *P. falciparum, P. vivax* and mixed infection | 1 | 3 | 0 | 4 |
| *P. falciparum* and *P. malariae* | 0 | 1 | 0 | 1 |
| *P. falciparum, P. vivax* and *P. malariae* | 0 | 1 | 0 | 1 |
| *P. vivax* and *P. malariae* | 0 | 1 | 0 | 1 |
| *P. vivax* only | 29 | 12 | 3 | 44 |
| Total | 41 | 85 | 9 | 135 |

**Supplementary Figure 1. Longitudinal antibody levels against six antigens for all women free from malaria (controls) by the serostatus to schizont extract.**


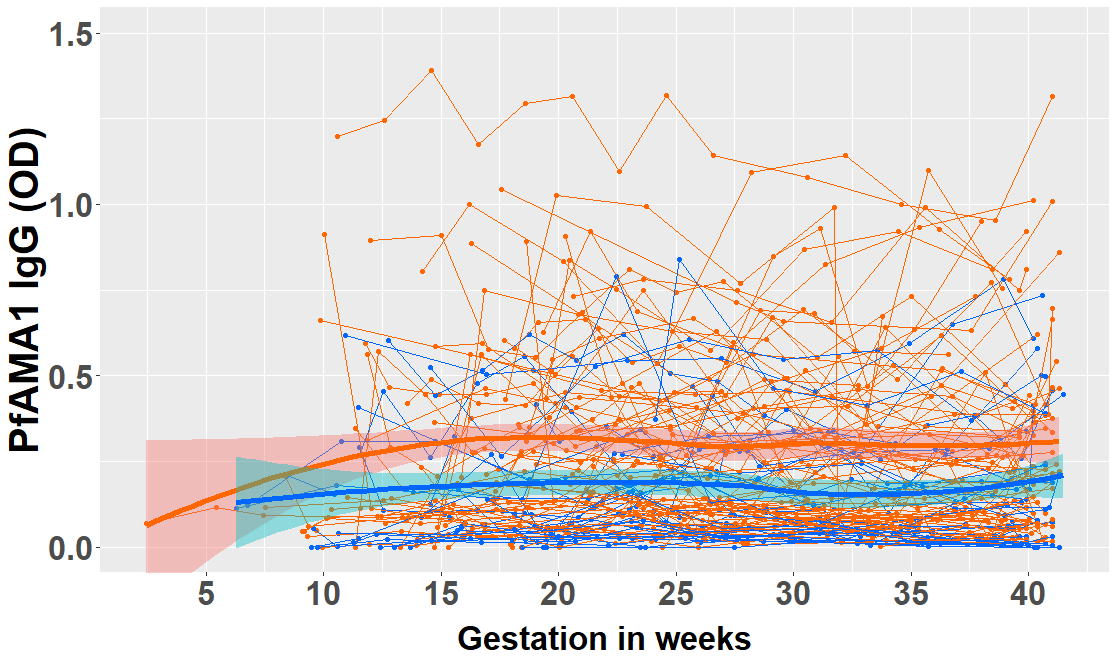

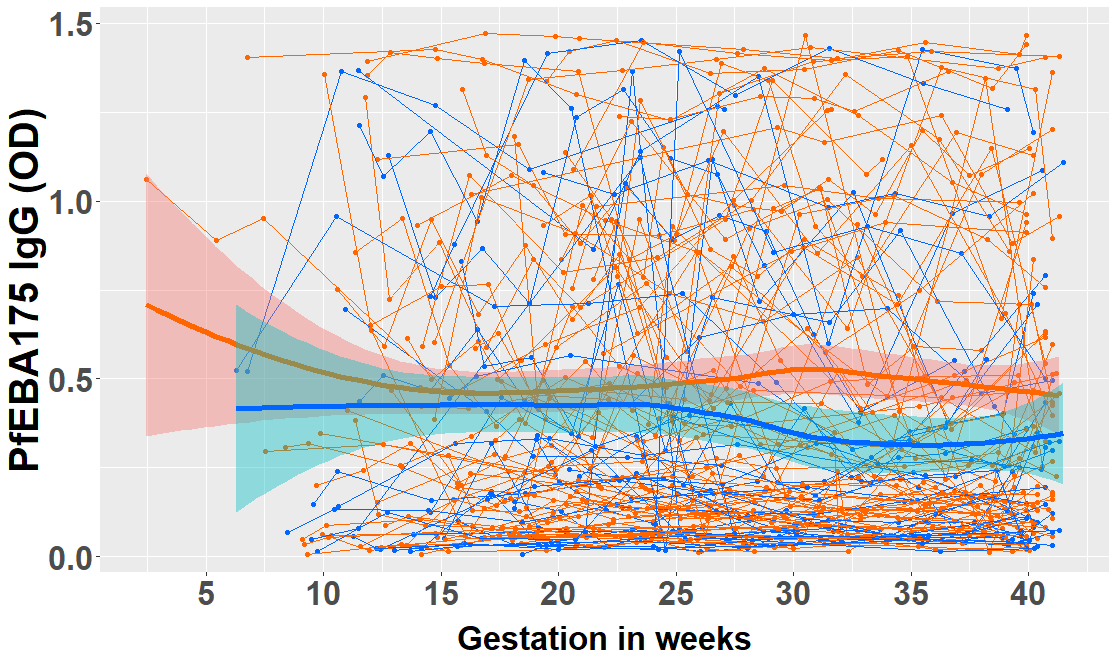

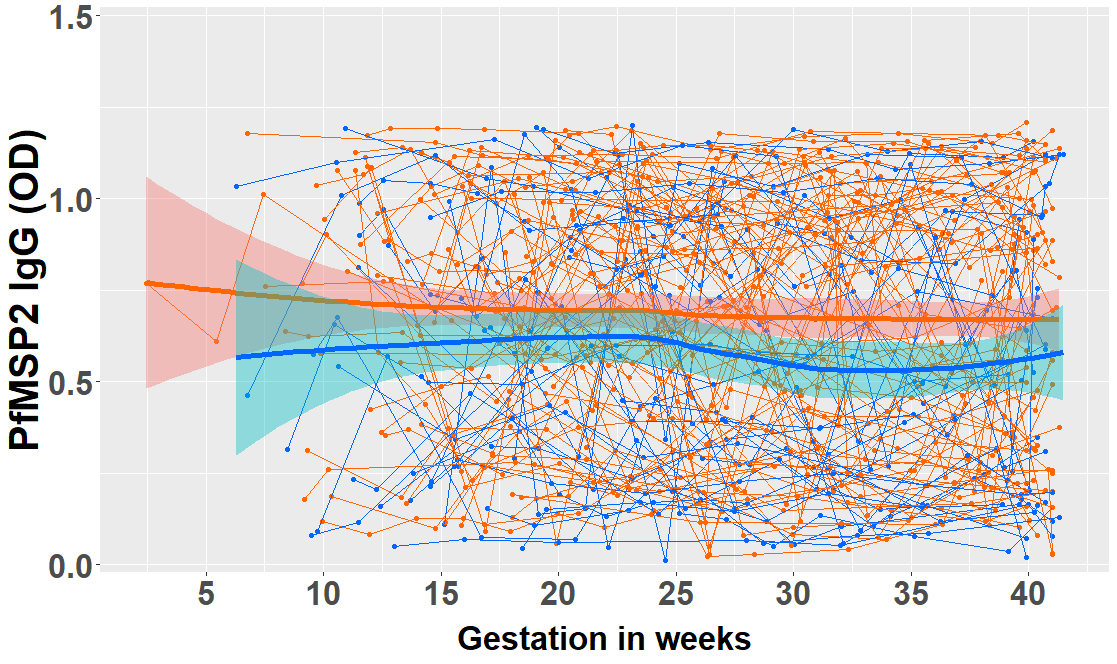

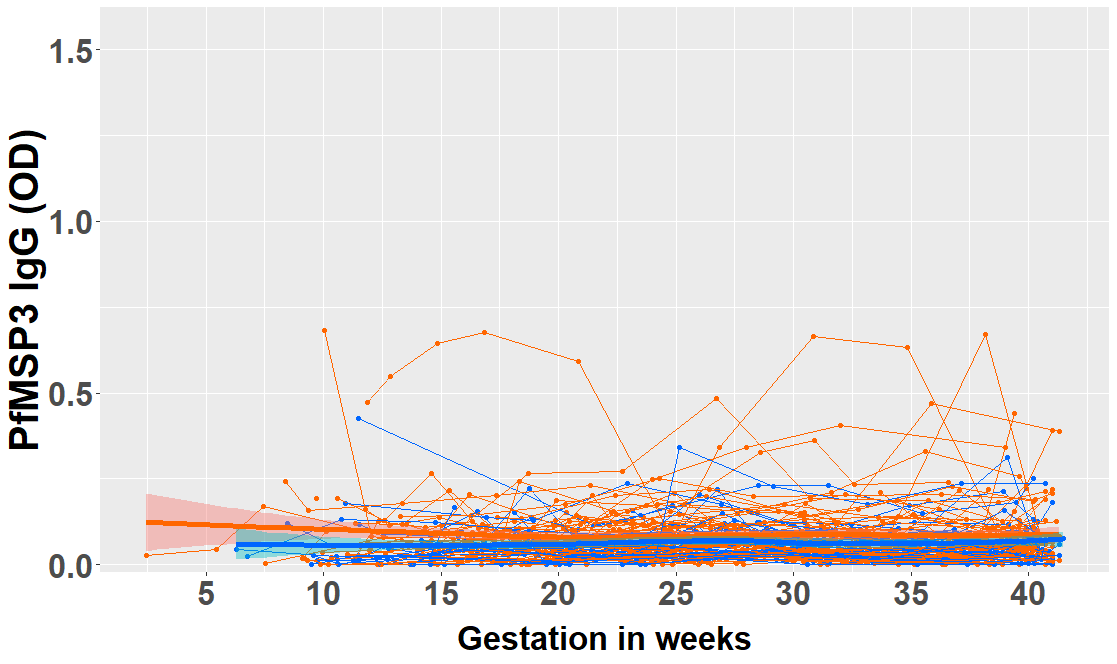

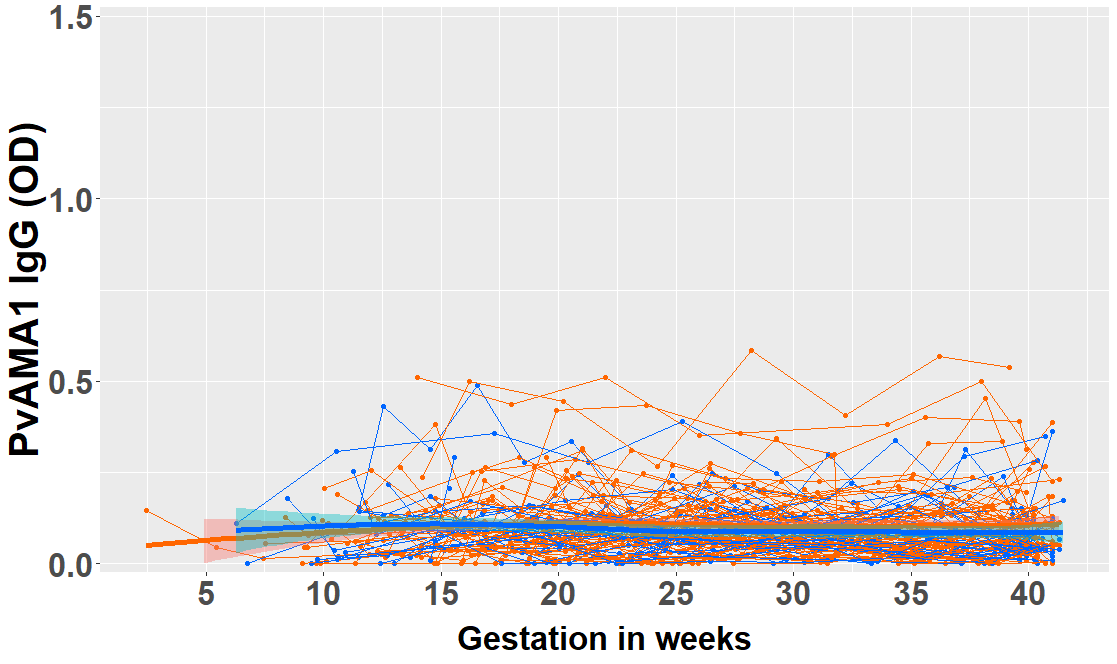

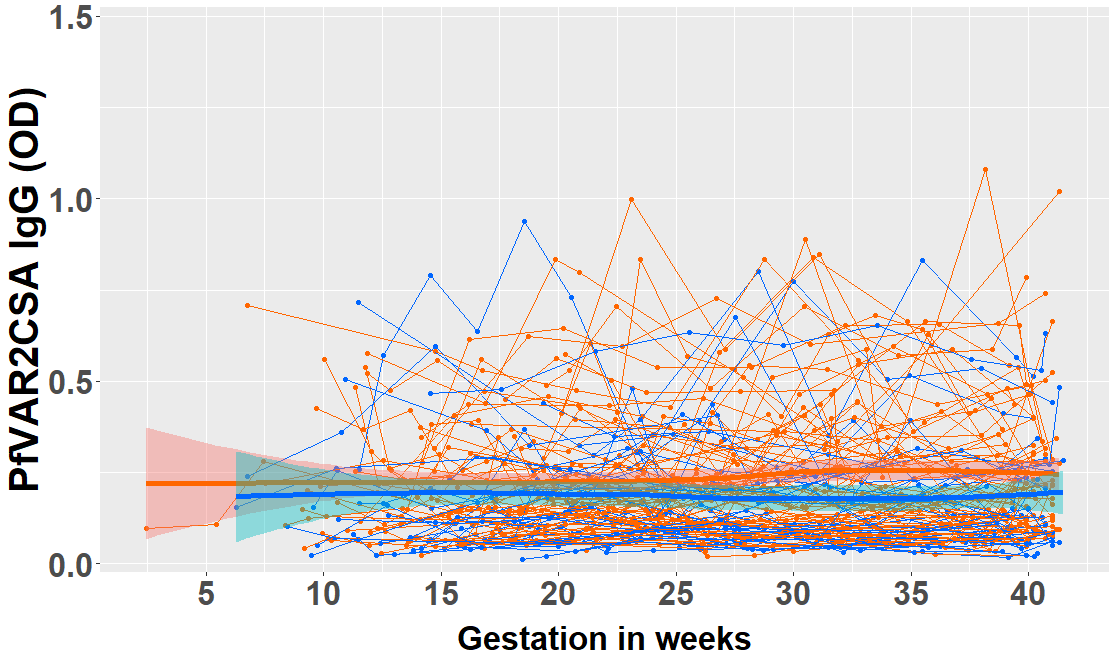


**A**

**B**

**C**

**D**

**E**

**F**

**Legend:** Spaghetti plots A–F represent the antibody profiles of *Pf*AMA1, *Pf*EBA175, *Pf*MSP2, *Pf*MSP3, *Pv*AMA1 and *Pf*VAR2CSA, respectively. The antibody levels of the controls seropositive and seronegative to schizont extract are represented by orange and blue, respectively. LOESS curves for each control subgroup are superimposed on each spaghetti plot. The shaded area around each LOESS curve represents the 95% confidence interval (CI). Of note, the Y axes of the plots of A and D are truncated at 0; the CIs did extend to negative values due to limited information in the early period of gestation. OD: Optical Density

**Supplementary Figure 2. Longitudinal antibody levels against six antigens for all women by gravidity (primigravidae versus multigravidae).**


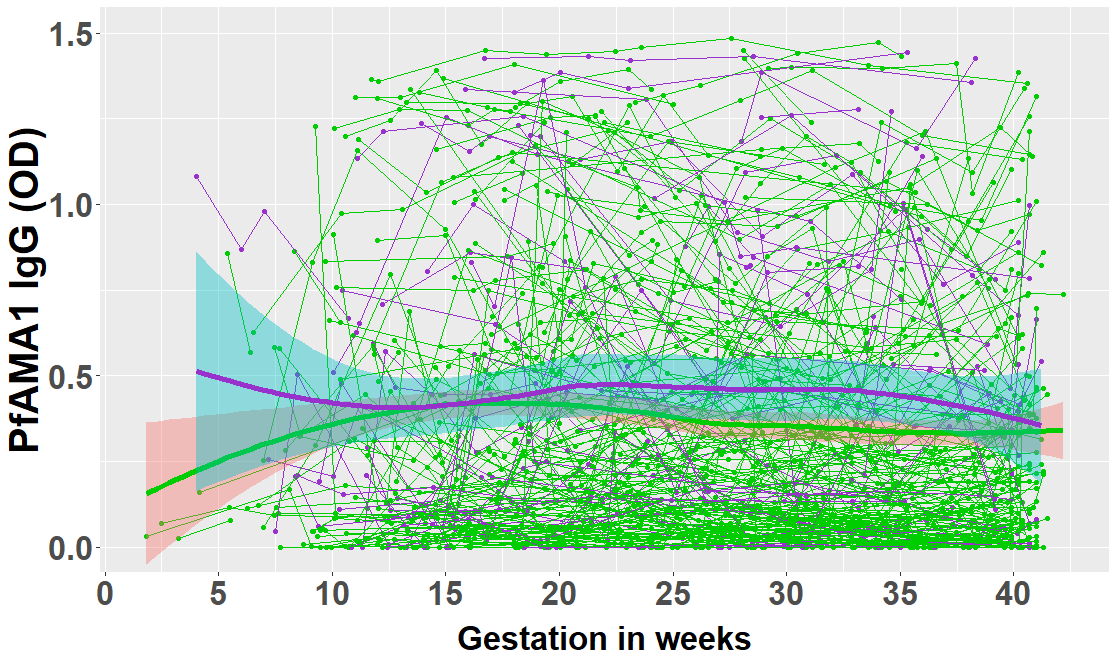

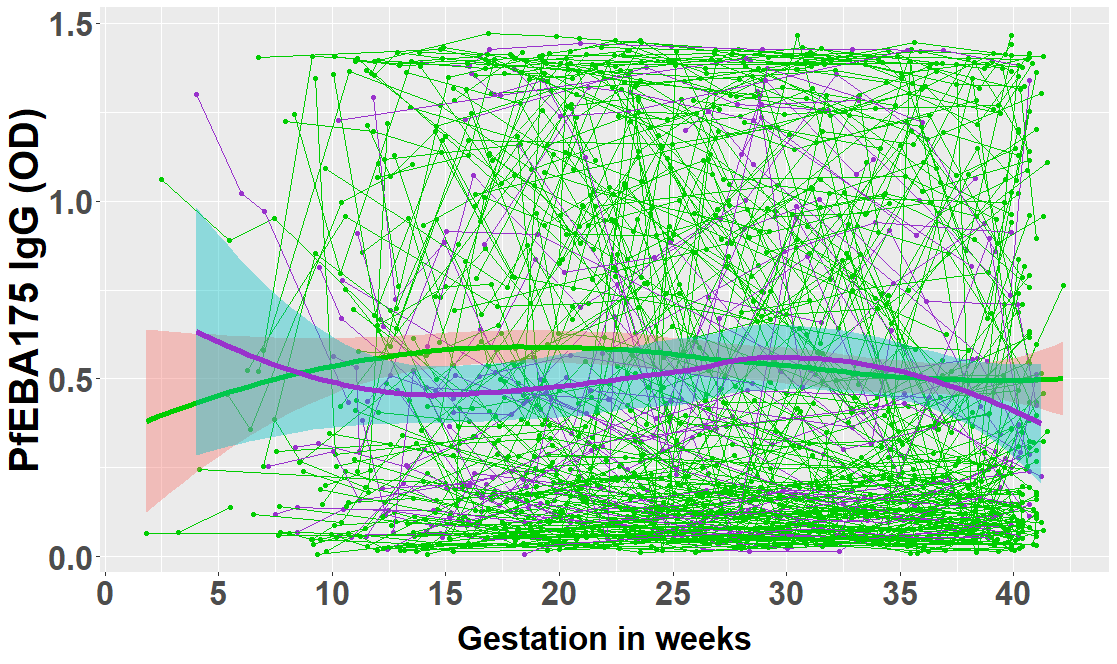

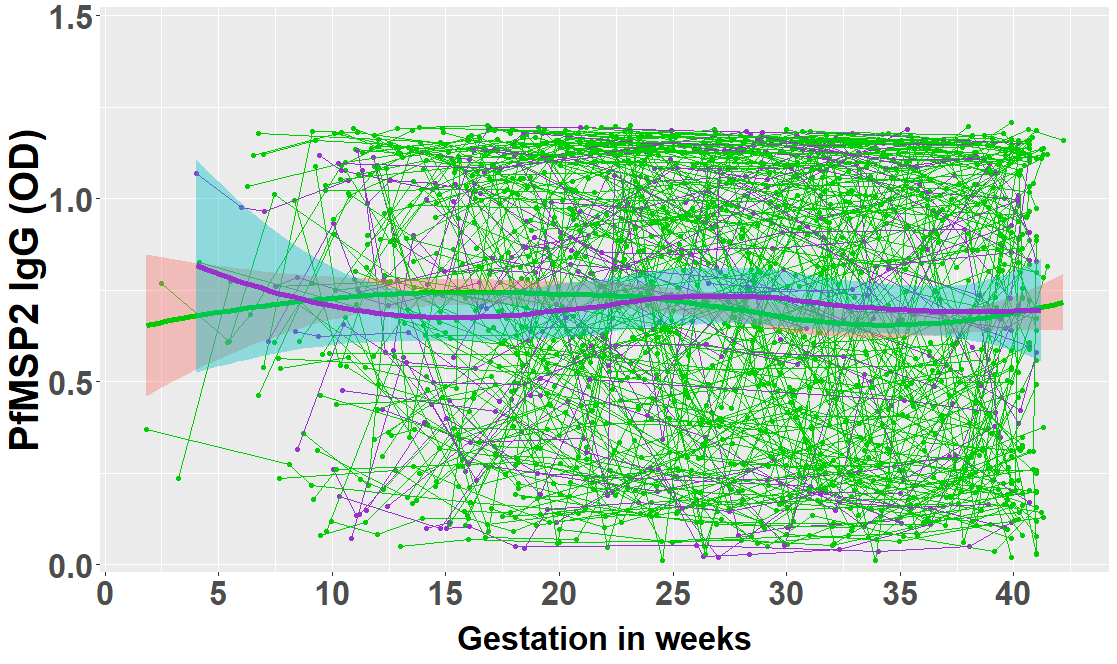

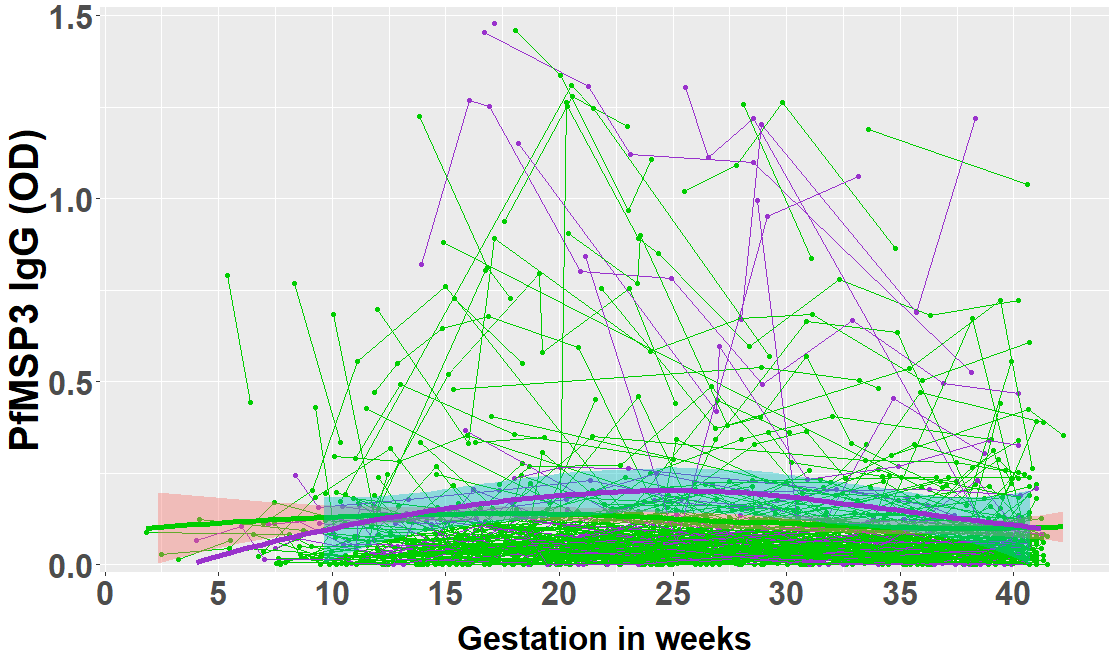

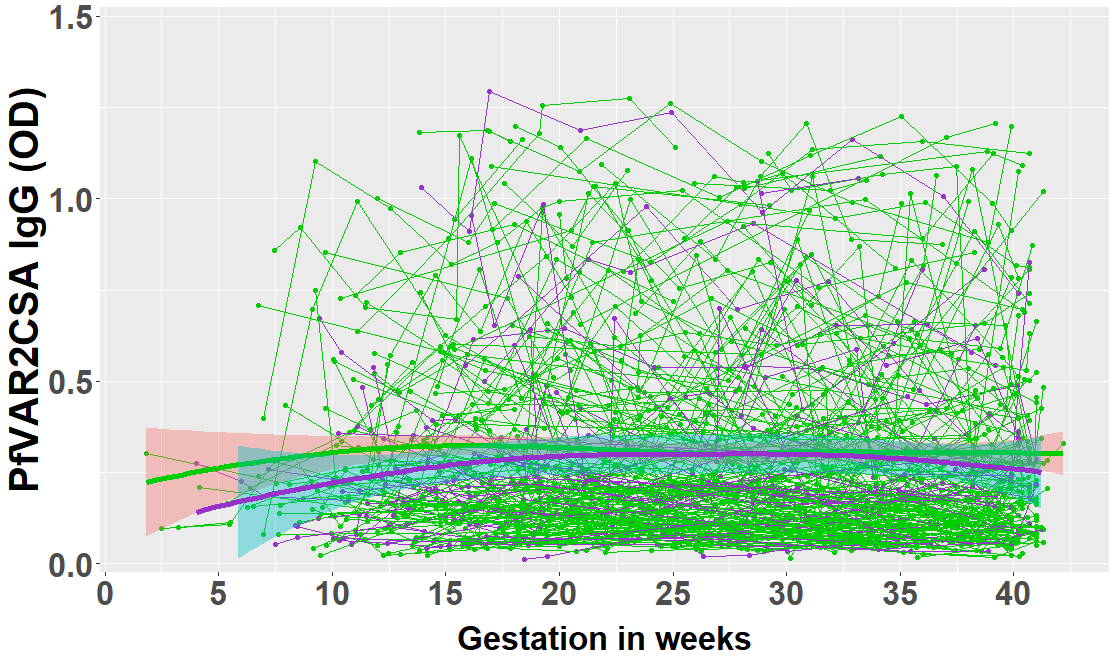

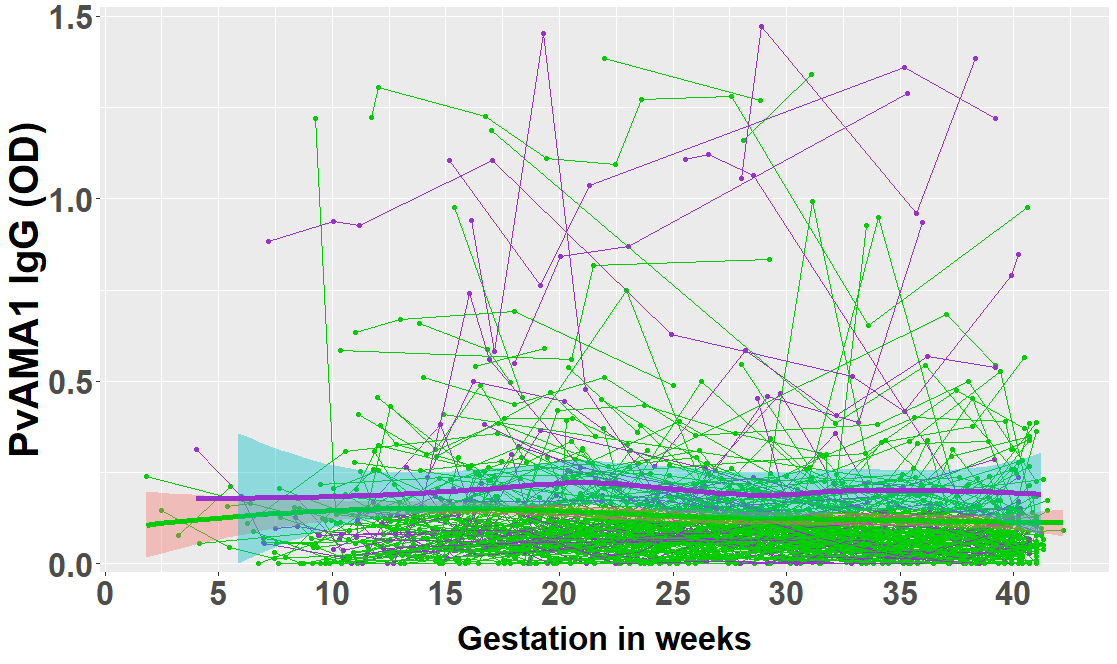


**A**

**B**

**C**

**D**

**E**

**F**

**Legend**: Spaghetti plots A–F represent the antibody profiles of *Pf*AMA1, *Pf*EBA175, *Pf*MSP2, *Pf*MSP3, *Pv*AMA1 and *Pf*VAR2CSA, respectively. The antibody levels of the primigravidae and multigravidae pregnant women are represented by purple and green, respectively.


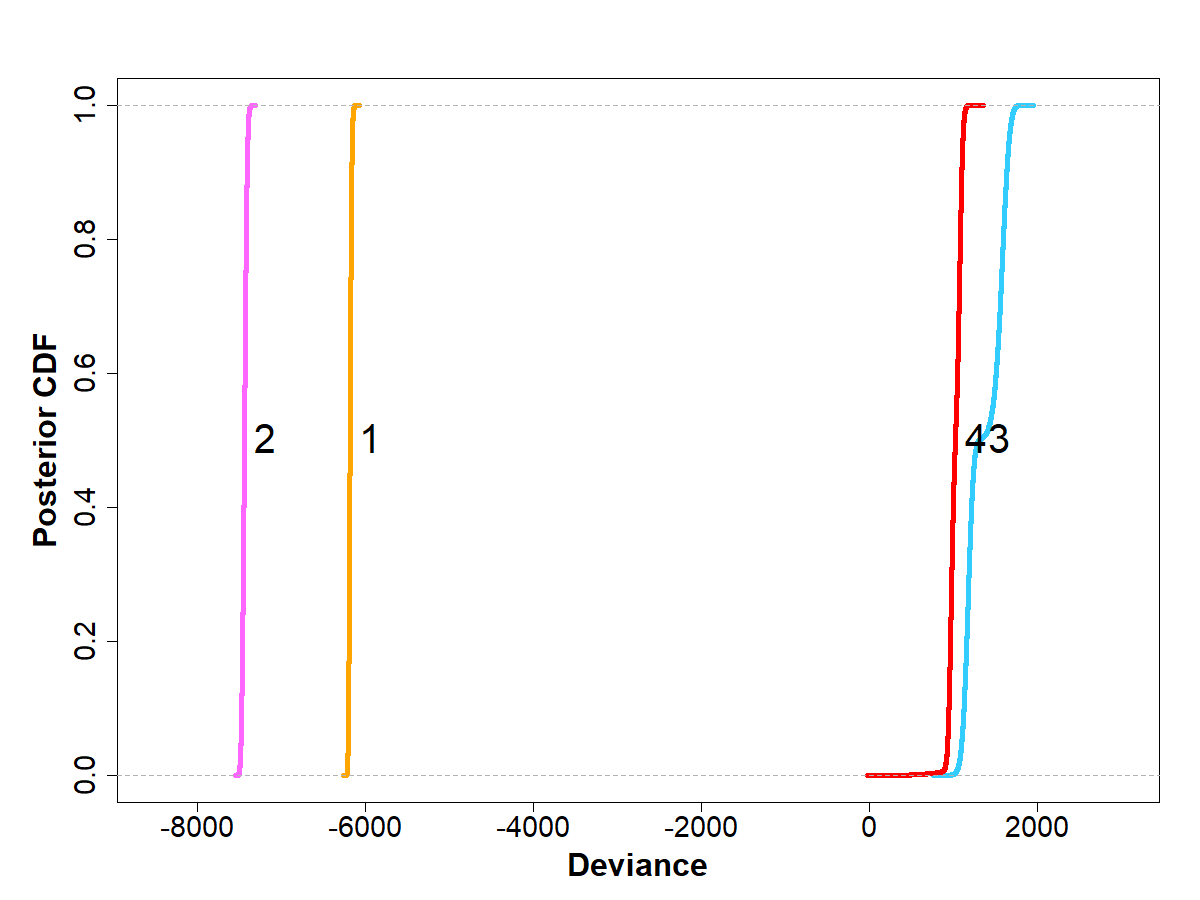
**Supplementary Figure 3. Posterior cumulative distribution functions of the observed data deviances for models with K = 1, 2, 3 and 4**

**Legend:** The posterior cumulative distribution of the deviance for the models with $K$ = 1, 2, 3 and 4 components are represented by orange, pink, blue and red line segments respectively.

The posterior cumulative distribution of the deviance for the model with $K$ = 2 components remained low throughout the 10,000 MCMC samples (remaining at the left corner of the plot). Hence in agreement with the PED value, it is optimal to classify the pregnant women into two clusters.

**Supplementary Material References**

1. Stanisic DI, et al. IgG subclass-specific responses against Plasmodium falciparum merozoite antigens are associated with control of parasitemia and protection from symptomatic illness. Infect Immun. 2009;77(3):1165-74.

2. Persson KE, et al. Variation in use of erythrocyte invasion pathways by Plasmodium falciparum mediates evasion of human inhibitory antibodies. The Journal of clinical investigation. 2008;118(1):342-51.

3. Komárek A, Komárková L. Capabilities of R package mixAK for clustering based on multivariate continuous and discrete longitudinal data. Journal of Statistical Software. 2014;59(12):1-38.

4. Richardson S, Green PJ. On Bayesian analysis of mixtures with an unknown number of components (with discussion). Journal of the Royal Statistical Society: series B (statistical methodology). 1997;59(4):731-92.

5. Fong Y, Rue H, Wakefield J. Bayesian inference for generalized linear mixed models. Biostatistics. 2010;11(3):397-412.

6. Komárek A, Komárková L. Supplement to “Clustering for multivariate continuous and discrete longitudinal data.”. 2013. doi:10.1214/12-AOAS580SUPP.

7. Stephens M. Dealing with label switching in mixture models. Journal of the Royal Statistical Society: Series B (Statistical Methodology). 2000;62(4):795-809.

8. Plummer M. Penalized loss functions for Bayesian model comparison. Biostatistics. 2008;9(3):523-39.

9. Aitkin M. Statistical inference: an integrated Bayesian/likelihood approach: Chapman and Hall/CRC; 2010.

10. Asparouhov T, Muthén B. Variable-specific entropy contribution. Retrieved from <http://www.statmodel.com/download/UnivariateEntropy.pdf>. 2014.
